# Validating genuine changes in heartbeat-evoked potentials using pseudotrials and surrogate procedures

**DOI:** 10.1162/IMAG.a.30

**Published:** 2025-06-10

**Authors:** Paul Steinfath, Nadine Herzog, Antonin Fourcade, Christian Sander, Vadim Nikulin, Arno Villringer

**Affiliations:** Department of Neurology, Max Planck Institute for Human Cognitive and Brain Sciences, Leipzig, Germany; International Max Planck Research School NeuroCom, Leipzig, Germany; Max Planck School of Cognition, Leipzig, Germany; Department of Psychiatry and Psychotherapy, University of Leipzig Medical Center, Leipzig, Germany; LIFE – Leipzig Research Center for Civilization Diseases, University of Leipzig, Leipzig, Germany; Department of Cognitive Neurology, University Hospital Leipzig, Leipzig, Germany; MindBrainBody Institute, Berlin School of Mind and Brain, Humboldt University Berlin, Berlin, Germany

**Keywords:** heartbeat-evoked potential, oddball task, pseudotrial correction, surrogate heartbeat analysis

## Abstract

The brain continuously receives interoceptive information about the state and function of our internal organs. For instance, each time the heart beats, the brain responds by generating time-locked activity, known as heartbeat-evoked potentials (HEP). When investigating HEPs, it is essential to adequately control for heartbeat-independent confounding activity to avoid false interpretations. In the present study, we highlight the pitfalls of uncontrolled analyses and advocate for the use of surrogate heartbeat analysis and pseudotrial correction, which are promising tools to control for spurious results. Surrogate heartbeat analysis involves shuffling the timing of heartbeats to verify the time-locking of HEP effects. Pseudotrial correction works by subtracting heartbeat-independent activity from HEPs. In this study, we employ both procedures, validate them in simulations, and apply them to real electroencephalography (EEG) data. Using EEG recordings obtained during the performance of an auditory novelty oddball task in a large population, we show that, without control analyses, pre-stimulus HEPs appear inversely related to task-related measures such as P300 event-related potential amplitudes and reaction time speed. However, these effects disappear after carefully controlling for heartbeat-unrelated EEG activity. Additionally, in real and simulated data, we show that pseudotrial correction has the potential to remove task-related confounds from HEPs, thereby uncovering real heartbeat-related effects that otherwise could be missed. This study, therefore, highlights issues that can arise when analyzing HEPs during tasks, provides solutions to overcome them, and gives recommendations for future studies to avoid pitfalls when analyzing and designing experiments involving HEPs.

## 1 Introduction

Interoception, the sensing of internal bodily states, is a fundamental aspect of physiological and psychological functioning. The brain constantly monitors and adjusts the state and function of our internal organs, including hunger, thirst, respiratory, and cardiac functions (Craig, 2002). Research has shown that the neural processing of visceral signals contributes to a wide range of emotional, perceptual, and cognitive processes (Azzalini et al., 2021;Critchley & Harrison, 2013;Quigley et al., 2021). Among these signals, the heart is a crucial source of interoceptive information. Neural responses to heartbeats can be directly measured using neuroimaging modalities such as electroencephalography (EEG) or magnetoencephalography (MEG). These heartbeat-evoked potentials (HEPs) are event-related potentials (ERPs) time-locked to participants’ heartbeats (Schandry et al., 1986).

HEPs reflect the cortical processing of afferent cardiac signals and are commonly used as an objective neurophysiological marker of cardiac interoception (Coll et al., 2021;Jones et al., 1986;Pollatos & Schandry, 2004;Schandry et al., 1986). Numerous studies have demonstrated that HEP amplitudes are significantly higher during interoceptive compared with exteroceptive attention (Montoya et al., 1993;Petzschner et al., 2019;Pollatos & Schandry, 2004;Pollatos et al., 2005;Schandry et al., 1986;Villena-González et al., 2017), suggesting that increases in its amplitude indicate an attentional shift toward internal stimuli (Al et al., 2020). While this relationship depends on the individual ability to feel the heartbeat (Yuan et al., 2007), it can be enhanced through training in cardiac perception (Canales-Johnson et al., 2015). Even though heartbeats are often not explicitly consciously processed, the brain nonetheless continuously generates HEPs, indicating often unconscious processing of heartbeat-related information (Kern et al., 2013;Perogamvros et al., 2019).

A critical challenge in HEP research lies in disentangling genuine cardiac-related neuronal activity from confounding processes that overlap temporally with heartbeat signals. For example, in tasks where subjects expect a stimulus requiring a motor response, a slow negative potential called contingent negative variation (CNV) can be present before the stimulus onset (Hillyard, 1969;Walter et al., 1964). If pre-stimulus HEPs are studied, it can happen that they temporally coincide with the CNVs and the overlap between the two neural signals can lead to false interpretations. Without adequate control analyses, the CNV-related activity might introduce heartbeat-unrelated effects (false positive) or mask genuine heartbeat-related HEP effects (false negative). Importantly, this issue is not specific to CNV, but can occur with any heartbeat-independent confounding activity that systematically differs between the conditions in which the HEPs are studied.

To address this issue, surrogate heartbeat control analyses have been suggested (Azzalini et al., 2019;Babo-Rebelo et al., 2016;Park & Blanke, 2019;Park et al., 2014,^2016^). This method operates on the premise that effects genuinely related to the heartbeat should be time-locked to it. To test for this time-locking, surrogate heartbeats are generated by perturbing the original timing of the HEP onsets (i.e., by randomly shifting the R-/T-peak markers in time (Park et al., 2016) or exchanging the heartbeat onset timing across trials (Azzalini et al., 2021)). This procedure is repeated many times, each time performing the same statistical analyses with the surrogate datasets. The results are then used to generate a null distribution to which the original HEP effects are compared. The main idea is that the heartbeat onset shuffling should preserve all other aspects of neuronal data not directly related to HEPs. If the original effect exceeds the effects obtained by analyzing surrogates, it likely reflects a genuine heartbeat-related process (Park et al., 2014).

However, a theoretical limitation of the surrogate heartbeat analysis is that genuine HEP effects may remain undetected if heartbeat-independent confounding factors, which are overlapping with the HEP, also differ between conditions (e.g., CNV, ERP components, oscillatory changes, or other processes). When shuffling the R-peaks in the surrogate analysis, the true HEP effect would disappear, because it is locked to the heartbeat. In contrast, the effects of heartbeat-independent confounders remain unaffected because they are not tied to the heartbeat timing. Consequently, the null distribution obtained from the surrogate heartbeat analysis would contain effects related to the heartbeat-independent confounder. Since we test the small but true HEP effect against the null distribution containing larger confounding effects, we are likely to fail to detect the HEP effect as statistically significant (Type II error).

In order to overcome this challenge, in this study, we thus combine a surrogate control analysis with a pseudotrial correction (Wainio-Theberge et al., 2021) to more effectively distinguish genuine HEP effects from confounding activity. Pseudotrial correction involves adding random “pseudo-R-peak” triggers to the same interval where the real heartbeats are studied. Hence, pseudotrials capture EEG activity which is unrelated to the heartbeat such as CNV, slow drifts, or other processes. By averaging and subtracting the pseudotrial from real HEPs, heartbeat-unrelated activity is removed while potentially revealing true heartbeat-locked responses.

In previous studies, HEP amplitudes were found to be higher for interoceptive attention than for exteroceptive attention (Al et al., 2020,^2021^;García-Cordero et al., 2017;Petzschner et al., 2019;Villena-González et al., 2017;Zaccaro et al., 2022,^2024^). However, the relationship between HEP amplitudes and attention-related task-evoked responses remains less well understood. Given that the P300 ERP component indexes an attentional shift toward external stimuli (Kok, 2001;Polich, 2007), we hypothesized to observe a negative relationship between HEP and P300 amplitudes. Additionally, since reaction times (RT) are related to on-task attention (Posner & Boies, 1971;Yamashita et al., 2021), we also hypothesized an inverse relationship with HEP amplitudes, with lower HEP amplitudes prior to faster reaction times. In line with our hypotheses, we initially observed a significant inverse relationship between the amplitudes of pre-stimulus HEPs and task-evoked P300 ERPs in an auditory novelty oddball task recorded in a large population-based EEG dataset (Loeffler et al., 2015). Furthermore, we also found the amplitude of HEPs to be lower before trials with faster RTs. While these observations align with the idea that the allocation of attentional resources toward exteroception has an influence on HEP amplitudes, the application of surrogate heartbeat and pseudotrial correction showed that these effects were likely spurious in our task. By utilizing resting-state as well as simulated data, we present alternative explanations that do not involve HEP-related effects.

Additionally, using both real and simulated data, we show that pseudotrial correction can uncover HEP effects which otherwise are masked by confounding factors, thereby reducing the chance of false negative findings. Our study underscores the need to apply careful control analyses when investigating the relationship between cardiac- and task-evoked activity and represents a significant advancement toward the robust examination of HEP effects. The suggested procedures provide an avenue for a more comprehensive understanding of the neurophysiological mechanisms through which HEPs may influence cognitive processes, and encourages a critical review of previous findings.

## 2 Methods

### 2.1 Participants

The data used in this study were recorded as part of the population-based LIFE-Adult study (Leipzig Research Center for Civilization Diseases, Leipzig University;Loeffler et al., 2015). Participants were chosen at random from the residence registration office, and provided their written informed consent. The participants received monetary remuneration for their participation. The study was approved by the University of Leipzig’s Medical Faculty’s ethics committee. EEG data were available from 2887 subjects. Only subjects with task and resting-state recordings were included. We excluded subjects with a history of brain hemorrhage, concussion, skull fracture, brain surgery, or brain tumor. No alcohol consumption was allowed on the day of the measurement. Participants who consumed central nervous system affecting medications were excluded (opioids, hypnotics and sedatives, anti-parkinsonian drugs, anxiolytics, anti-depressants, anti-psychotics, anti-epileptic drugs). In addition, subjects with less than 20 heartbeats in the pre-stimulus time of the target stimuli were excluded (seeSections 2.3and2.5). After applying these criteria, our final sample included 1739 participants’ datasets (mean age = 70, SD = 4.6, 874 females).

### 2.2 EEG recording

EEG data were recorded from 31-channel Ag/AgCl scalp electrodes (Brain Products GmbH, Gilching, Germany) in an electrically shielded and soundproof EEG booth. The electrodes were mounted in an elastic cap (easyCAP, Herrsching, Germany) according to the international standard 10–20 extended localization system. The signal was amplified with a QuickAmp amplifier (Brain Products GmbH, Gilching, Germany). Additionally, two electrodes recorded vertical (vEOG) and horizontal (hEOG) eye movements above and beneath the right eye. One bipolar electrode attached to the right and left forearm recorded electrocardiogram (ECG). The electrodes were referenced to the common average reference, with AFz being a ground electrode. The electrodes’ impedances were kept below 10 kΩ, the sampling rate was 1000 Hz, and the data were low-pass filtered at 280 Hz. At the start of the EEG measurement, the participants underwent a 20-min-long resting-state EEG (rsEEG) measurement. During this time, they laid on their back in a dark room and closed their eyes. The participants were instructed to avoid falling asleep. This was followed by a 15-min-long auditory novelty oddball paradigm described below (Section 2.4). A more detailed description regarding the recording can be found in a paper byJawinski et al. (2017).

### 2.3 EEG data processing

EEG processing was performed with MATLAB (version R2024a) using custom-written scripts as well as the EEGLAB (Delorme & Makeig, 2004), Fieldtrip (Oostenveld et al., 2011), HEPLAB (Perakakis, 2019), and ERPLAB (Lopez-Calderon & Luck, 2014) toolboxes. EEG data were filtered with a 4th order Butterworth filter, first high pass with a 1 Hz cutoff, and subsequently low pass with a 45 Hz filter cutoff frequency. In addition, to reduce residual line noise, a notch filter between 49 and 51 Hz was applied using the EEGLAB pop_eegfiltnew function. The data were down sampled to 250 Hz. Bad channels were identified and removed using the clean_artifacts EEGLAB plugin if they were flat for more than 5 s, correlated with their neighbors less than 0.85, or had residual line noise (average number of removed channels 0.94,*SD*= 1.15). Removed channels were interpolated using spherical interpolation. To identify noise sources in the data, we performed Independent Component Analysis (ICA) using the extended infomax algorithm after Principal Component Analysis based dimensionality reduction to the rank of the data. R-peaks were identified in the ECG data using the heplab_slowdetect function (Perakakis, 2019). ECG and independent component (IC) time courses were segmented between -0.05 and 0.6 s around the R-peak markers, averaged across epochs, and correlated with each other. ICs with a correlation greater than 0.8 with the ECG were marked as artifacts (Zaccaro et al., 2024). IClabel was used to identify potential components related to eyes (prob. ≥ 0.6), muscles (prob. ≥ 0.5), line noise (prob. ≥ 0.5), channel noise (prob. ≥ 0.4), and other (prob. ≥ 0.5). The automatically marked components were reviewed manually, and the selection adapted if necessary. An average of 1 heart-related ICs (*SD*= 1.08, min 0, max 5; average explained variance: 5.47%) were removed from the data. The same preprocessing steps up until the ICA decomposition were repeated on the same data, but with a high-pass filter cutoff of 0.3 Hz. The ICA weights were copied to these data, and components marked as artifacts were removed. This was done, since ICA performs better with a higher high-pass filter cutoff (Winkler et al., 2015), while slow ERP components could be degraded at such (Tanner et al., 2015).

### 2.4 Oddball task

**Fig. 1.**
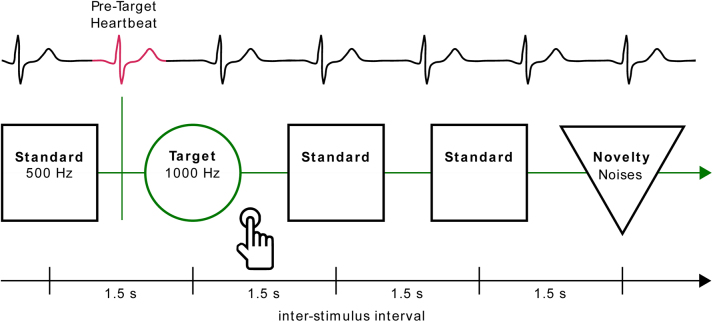
Schematic of the Auditory Novelty Oddball Task. Participants performed a 15-min-long auditory Novelty Oddball task in which they were presented with standard (500 Hz), target (1000 Hz), and novelty (environment or animal) sounds with a stable inter-stimulus interval of 1.5 s. They were instructed to press a button each time the target stimulus occurred. Heartbeats in the pre-stimulus time of target trials were identified and used as markers for time-locked pre-stimulus heartbeat-evoked potentials.

A novelty auditory oddball paradigm was used to elicit auditory event-related potentials (Fig. 1). In total, 600 stimuli were presented in a pseudo-randomized order with a minimum of two standard stimuli between target stimuli, a maximum of nine standard stimuli in succession and a constant inter-stimulus interval (ISI) of 1500 ms. The more frequent, non-relevant standard stimuli (500 Hz sinusoidal tone lasting 40 ms including 10 ms rise and fall times) occurred 456 times with a global probability of 76%. Both the task-relevant target (1000 Hz sinusoidal tone lasting 40 ms including 10 ms rise and fall times) and novelty stimuli (environment or animal sounds lasting 400 ms with variable rise and fall times) occurred 72 times with a global probability of 12% each. The participants were instructed to press a button when the target stimulus occurred. After 300 stimuli, the paradigm was interrupted by a short pause (30 s), in which the participant was asked to change the response hand. For the current study, we only analyzed target stimuli and their preceding HEPs. The participants performed the task with an accuracy of 96.23% correct trials, 1.3% incorrect responses, 2.45% omission errors, and an average reaction time of 489.6 ms.

### 2.5 Heartbeat-evoked potentials

We only selected pre-stimulus HEPs that occurred within the time window of -1100 and -600 ms before the target onset, in order to prevent interference from the preceding stimulus or the evoked response related to target stimulus presentation. HEPs were epoched between -200 and 600 ms around the R-peak. Epochs that exceeded a peak-to-peak signal amplitude ≥150 µV in any channel using a moving window (window size = 200 ms, window step = 100 ms) were discarded. If fewer than 20 pre-stimulus HEPs were found, the subject was excluded from further analysis. As a result, on average, 27.2 (*SD*= 4.5) HEPs were included per subject. Baseline correction was applied based on the -150 to -50 ms time window. Furthermore, due to the repetitive nature of the heartbeat, there is no ideal baseline window that is free of all activity (Azzalini et al., 2019,Babo-Rebelo et al., 2016;^2019^;Banellis & Cruse, 2020;Park et al., 2014;Verdonk et al., 2021). Hence, we repeated the same analyses without baseline correction and included the results in the Supplementary Materials (Supplementary Figures 1–6andSupplementary Table 1). To assess whether the use of baseline correction could introduce unwanted biases, we also included the baseline interval in the cluster-based permutation tests (seeSection 2.8).

### 2.6 ERP analysis

Target trials were epoched between -500 and 1000 ms around the stimulus onset. Epochs that exceeded a maximum moving window peak-to-peak signal amplitude >=150 µV were removed (window size = 200 ms, window step = 100 ms). To enhance the single-trial signal-to-noise ratio, we utilized a spatial filter derived from linear discriminant analysis (LDA) to distinguish between target and standard stimuli (Blankertz et al., 2011). For each participant, the average amplitude within the time window from 200 to 700 ms was calculated for target and standard ERPs, which yielded two amplitude values per participant. Using the amplitude values as features, we trained an LDA model to classify between the target and standard ERPs. The LDA model learns to maximize the difference between the two classes while minimizing the variance within each class. As a result, we obtain weights from the LDA model which reflect a spatial filter, which can be applied to the single trial data of each participant, resulting in a single P300 ERP time course per trial. To investigate how pre-stimulus HEPs relate to cognitive functioning, we sorted pre-stimulus HEPs into (a) high versus low P300 trials based on a median split of single trial average ERP amplitude within the 250–600 ms time window (average amplitude cutoff: 3.26 μV) and (b) in fast and slow category based on a median split of the subjects’ reaction times (RT, average cutoff: 476 ms). No baseline correction was applied for the P300 target trials, as subtraction of pre-stimulus from post-stimulus amplitudes creates a dependency between the two. Since the HEPs we investigate are in the pre-stimulus time of the P300 ERP trials, this dependency might then be reflected in the amplitude of pre-stimulus HEPs, ultimately confounding the analysis.

### 2.7 Control analysis for P300 sorting using resting-state data

We performed a control analysis to determine whether HEP effects observed after sorting stimuli into high and low P300 conditions were uniquely tied to the task or could emerge from the data-sorting approach itself. Specifically, we copied the trial structure from the oddball task to the resting-state recordings of the same participants. This way, no genuine task-evoked activity was present, while real heartbeats and HEPs remained. We then repeated the same sorting procedure used for the real P300 ERP data: dividing these resting-state trials into high- and low-amplitude groups (“pseudo-ERPs”) based on their amplitude in the 250–600 ms post-stimulus time range. Finally, cluster-based permutation*t*-tests were used to assess whether pre-stimulus HEP differed with respect to the high and low “pseudo-ERP” conditions.

### 2.8 Statistical analyses

We performed cluster-based permutation*t*-tests (CBP, two-tailed) on the data using the FieldTrip toolbox (Maris & Oostenveld, 2007) to compare HEPs between our conditions (HEPs in the pre-stimulus time of: high vs. low P300, high vs. low “pseudoERP”, and fast vs. slow RT). The cluster-based permutation procedure is a method used to identify significant effects in time and space while accounting for multiple comparisons. This method works by adding up the*t*-values of all significant tests (*p*-value of <0.05) that are adjacent in both space and time. These experimentally observed cluster-specific*t*-values are compared with a permutation distribution that is generated by randomizing the condition labels (in our case 1000 times) each time selecting the maximum summed*t*-value across all clusters. Clusters with a*p*-value smaller than an adjusted alpha level of 0.05 were considered significant. The precision of*p*-values derived from permutation tests is limited by the number of permutations. Since we applied 1000 permutations, the minimum*p*-value is 0.001. As previous studies are diverse in their temporal and spatial HEP findings, we included all 31 channels and the time window of -200 to 600 ms around the R-peak in our analyses. To control for volume conducted ECG artifacts, we compared the ECG signal related to the different conditions by temporal cluster-based permutation*t*-tests.

### 2.9 Surrogate heartbeat control analysis

**Fig. 2.**
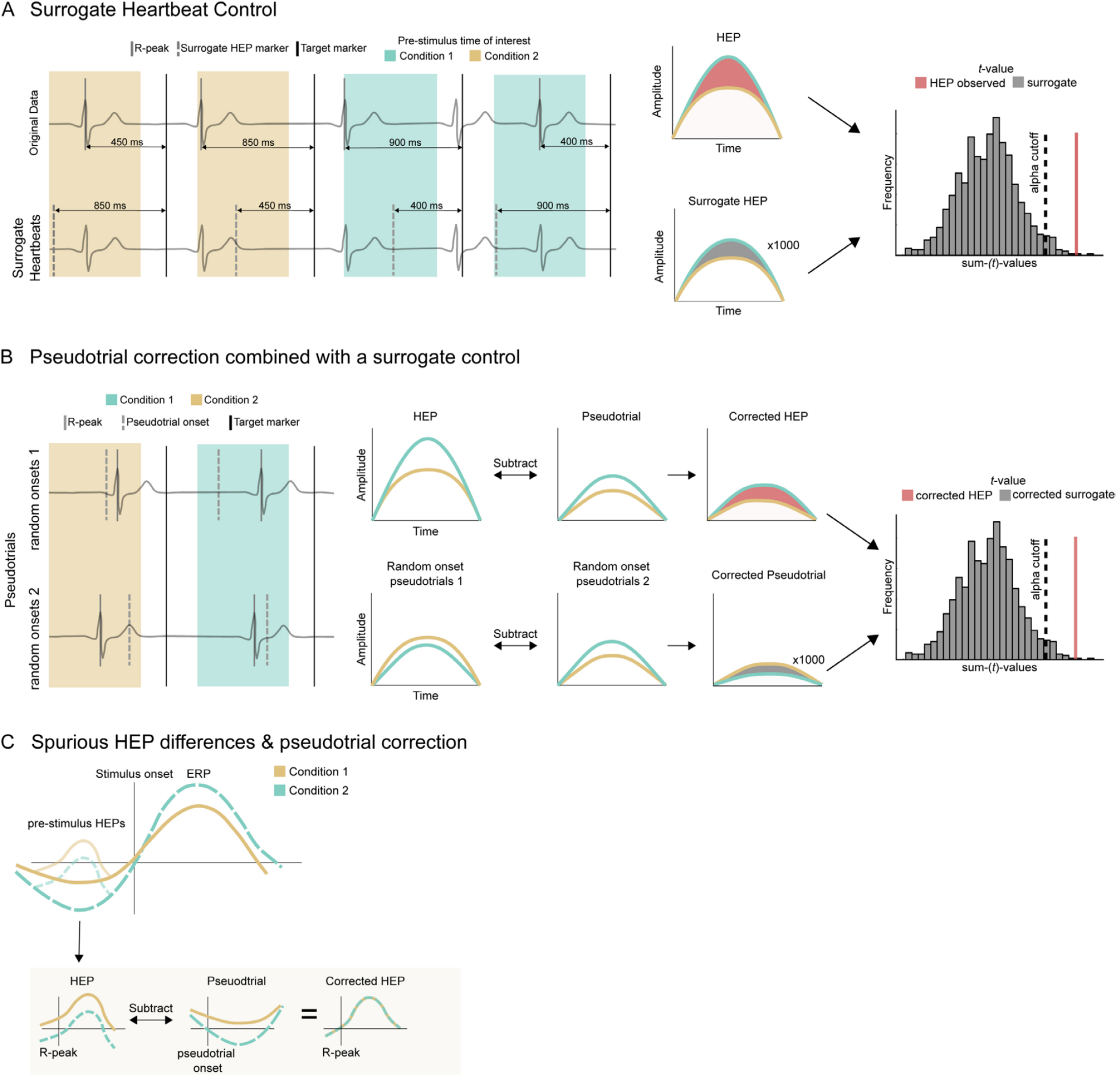
Schematic overview of the main methods used in this study. (A) Illustration of the surrogate heartbeat procedure adapted in this study. HEPs are calculated based on heartbeats occurring the pre-stimulus time of two different conditions. To control for heartbeat unrelated effects, R-peak timings relative to the target stimuli are shuffled within each condition, generating surrogate HEPs. This procedure is repeated a number of times, each time calculating the condition difference between surrogate HEPs. The resulting null-distribution of cluster sum(*t*)-values is used to assess the significance of the original HEP effect. (B) Pseudotrials are generated by adding random triggers to the pre-stimulus time of interest. HEPs are corrected by subtracting pseudotrials per condition, potentially revealing some residual effect. To control for effects introduced by the pseudotrial correction, different permutations of random pseudotrials are subtracted from each other per condition. This procedure is repeated a number of times, each time calculating the condition difference between the corrected pseudotrials. The resulting null-distribution of cluster sum(*t*)-values is used to assess the significance of the original HEP effect. (C) Schematic representation of spurious HEP differences based on heartbeat-unrelated condition differences. Subtraction of pseudotrials removes unrelated activity.

To ensure that the observed HEP effects are genuinely linked to heartbeats and not a result of changes in ongoing spontaneous activity, we conducted a surrogate heartbeat analysis (Fig. 2A;Babo-Rebelo et al., 2016). As a first step, we shuffled the pre-stimulus R-peak onsets within high & low P300 (fast & slow RT) condition. Then we performed the same statistical analysis using cluster-based permutations, while saving the absolute summed*t*-value of the largest identified cluster. Since in the surrogate data the temporal relationship between R-peak and HEP was broken by permuting the R-peak timings, the identified clusters should reflect heartbeat-unrelated processes. Since R-peak latencies were shuffled within subject and across conditions, the mean heart-rate and inter-beat interval are kept the same. We repeated this procedure >100 times, each time shuffling the pre-stimulus R-peak timings. At the end, we compared the absolute cluster sum(*t*)-values from the original data with the corresponding distribution of the surrogate statistics. The original*t*-value has to be larger than 95% of the surrogate cluster sum(*t*)-values to conclude a significant effect. While both, the surrogate heartbeat control analysis and CBP, involve permutation approaches, they serve fundamentally different purposes. CBP tests the null hypothesis that HEP effects are unrelated to the different experimental conditions (i.e., no differences between the conditions) by shuffling the condition labels across trials (seeSection 2.8). In contrast, the surrogate heartbeat procedure tests if HEP effects are time-locked to heartbeats by shuffling R-peak timings within each condition. This disrupts the temporal alignment between heartbeat and EEG activity while preserving any heartbeat-unrelated but condition-specific activity. When comparing the statistics of the original (non-shuffled) data with the distribution obtained from surrogate data, we test the null-hypothesis that the observed HEP effects are not specifically time-locked to the heartbeats.

### 2.10 Pseudotrial correction

If genuine HEP effects overlap with non-heartbeat-related processes (e.g., slow drifts, preparatory activity), the true HEP effects can be overshadowed by the heartbeat-unrelated processes and remain undetected (type II error, see results of data simulation part 2.12). To uncover potentially present genuine HEP effects, we adopted a pseudotrial correction method suggested earlier for other purposes (Fig. 2B, C;Wainio-Theberge et al., 2021), which removes heartbeat-independent effects. In this method, activity that is present in the pre-stimulus time of trials independent of a heartbeat is subtracted from real HEPs per condition, thereby effectively controlling for heartbeat-independent condition differences and data sorting-related regression to the mean effects (Bland & Altman, 1994). We implemented the pseudotrial correction in the following way: for each target trial, a pseudo-R-peak-trigger was inserted at a random time point in the pre-stimulus time of interest (-1100 to -600 ms). Second, the data were sorted according to high and low P300 amplitude or fast and slow RT and averaged within these categories, resulting in pseudotrials. Lastly, within each subject, the real HEP epochs are corrected by subtracting the pseudotrials per condition.

### 2.11 Equivalence test

For non-significant results, we employed equivalence testing to assess whether the difference between conditions was smaller than a practically meaningful effect (smallest effect size of interest, SESOI). In contrast to classical null hypothesis testing, where the hypothesis is tested that a difference between groups exists, in equivalence testing the hypothesis is tested that the group difference is smaller than a SESOI. Hence, it is critical to define a reasonable SESOI which serves as the bound for effects to be considered too small to be meaningful (Lakens et al., 2018). Our HEP analyses are based on the idea that their amplitudes are modulated based on spontaneous fluctuations of interoceptive versus exteroceptive attention. Hence, we chose to inform our SESOI by meta-analytic results fromColl et al. (2021)who investigated the relationship between HEP amplitudes and interoception across various publications in different domains (attention, performance, clinical, and arousal). From all reported effects across the different domains, we use the lowest end of the lowest confidence interval, which represents a Hedge’s*g*= 0.19 (performance category). Hence, we defined our SESOI as an effect smaller than a Hedge’s*g*of 0.19 and larger than -0.19. We used the two one-sided tests (TOST) procedure implemented in the R package TOSTER (version 0.8.0,Caldwell, 2022;Lakens, 2017).

### 2.12 Data simulation

To illustrate the increased power to detect effects when a pseudotrial correction is applied, we simulated data containing interactions between simulated HEP (simHEP) and task ERP (simERP). As a background signal with characteristic close to real data, we used EEG data of 100 random subjects from the Pz electrode which was scrambled by phase randomization to remove genuine activity (Gias, Carlos, 2024;Theiler et al., 1992). Phase randomization was achieved by first calculating the fast Fourier transform (FFT) of the original time-series signal, randomizing the phases, and performing the inverse FFT to reconstruct time-series data. As a result, genuine evoked responses (ERP and HEP) are removed from the signal, while oscillations at all frequency ranges remain intact.

Task triggers were added to this scrambled data every 4 s. We then simulated both positive and negative relationships between simHEP and simERP amplitudes with different magnitudes. This was achieved by first generating realistic normally distributed simERP amplitudes (mean = 7 µV,*SD*= 2) and simHEP amplitudes (mean= 1.5 µV,*SD*= 0.8). The signal-to-noise ratio (SNR, ratio of mean power between simulated evoked responses to the power of the phase randomized background signal) was 3.3 dB for simERPs and -9.3 dB for simHEPs. A scaling factor (k) between 0 and 1 for positive or 0 and -1 for negative relationships was used to represent the relationship strength between the two variables. To obtain simHEPs which are related to the simERP, we used the following approach: the normalized simERP amplitude was multiplied by the scaling factor adding the product of the square root of (1 – k²) and normalized simHEP amplitude. The resulting simHEP amplitudes were rescaled by their standard deviation and mean.

After generating correlated simERPs and simHEPs, they were modeled as positive deflections based on Gaussian functions in a single channel. The simERP onset was at 300 ms post-stimulus (duration 600 ms), and for half of the trials a pre-stimulus simHEP starting at 200 ms post-stimulus (duration of 400 ms) was added at a random time between -1500 and -600 ms relative to the stimulus onset. Pseudotrials were defined as random triggers in the same pre-stimulus time of trials without simHEPs. On average, 421 trials (*SD*= 45) were present per simulated dataset.

We sorted the simERP trials based on their amplitude in the 250–600 ms time range, and tested for significant differences between the pre-stimulus HEPs using cluster-based permutation testing in the temporal domain. We then tested whether a significant difference can be found after simHEPs were corrected by pseudotrial subtraction. A surrogate heartbeat control analysis was performed by randomly exchanging the onsets of pre-stimulus triggers per high and low amplitude condition before performing a cluster-based permutation test.

To adapt the surrogate procedure for pseudotrial corrected data, pre-stimulus triggers were randomly exchanged twice per high and low amplitude condition generating two sets of pseudotrials per condition. Next, the pseudotrials were averaged and subtracted from each other per condition, while the remaining activity was compared using cluster-based permutation testing. In this way, we control for insufficiently removed artifacts or potentially introduced noise by the pseudotrial correction procedure itself. This procedure was performed for each of the relationship directions (positive and negative association between pre-stimulus HEP and P300 ERP) and strength, and repeated 500 times to get a distribution of observed effects (maximum cluster-sum(*t*)) in each of the conditions.

Formally, the steps can be described as follows.

Generate normally distributed simERP and simHEP amplitudes:

$$A_{simERP}∼N\left(7,2^{2}\right),A_{simHEP}∼N\left(1.5,0.8^{2}\right)$$

AsimERP and AsimHEP are the simulated ERP and HEP amplitudes. The notation$N(\mu ,\sigma^{2})$represents a normal distribution with a meanμand variance$\sigma^{2}$.

Next, normalize simERP and simHEP amplitudes:

$${\hat{A}}_{simERP}=\frac{A_{simERP}-\mu_{simERP}}{\sigma_{simERP}},{\hat{A}}_{simHEP}=\frac{A_{simHEP}-\mu_{simHEP}}{\sigma_{simHEP}}.$$

Calculate simHEPs which are related to simERP:

$$A\prime_{simHEP}=k\cdot {\hat{A}}_{simERP}+\sqrt{1-k^{2}\cdot }{\hat{A}}_{simHEP},$$

where k is a scaling factor between 1 and 0 for positive and -1 and 0 for negative relationships.

Rescale the resulting simHEP amplitudes:

$$A{\prime \prime }_{simHEP}=A\prime_{simHEP}\;\cdot \;\sigma_{simHEP}+\mu_{simHEP.}$$

### 2.13 Δ ECG with Δ EEG correlation analysis for cardiac field artifact influence

While there was no significant ECG difference between the investigated task conditions, we did observe significantly different ECG amplitudes between resting-state and task recordings (Fig. 8C). Hence, it is possible that the observed HEP effects are explained by differences in cardiac field artifacts (CFA). CFA refers to the electric field generated by the heart which propagates through the body and is picked up by the EEG electrodes. To investigate the potential influence of the CFA on HEPs when comparing resting-state and task recordings, we assessed whether the difference between the two conditions in ECG (ΔECG)correlates with the difference in EEG (ΔEEG). Across all subjects, the correlation betweenΔECGandΔEEGwas assessed by Spearman correlation for each time point and electrode. Next, we identified clusters of significant correlations comprising a minimum of two neighboring channels. Surrogate statistics were calculated based on the correlation of randomly permutedΔECGtime courses with theΔEEG. This was the procedure repeated 1000 times with shuffledΔECG, each time retaining the summed*t*-value of the largest cluster. Lastly, we compared the summed*t*-values of each empirical cluster with the distribution of maximum summed*t*-values across all permutations. Clusters were considered significant if their summed*t*-value was larger than 95% of the summed*t*-values found in the surrogate analysis.

## 3 Results

### 3.1 HEP differences precede ERPs with high and low P300 amplitude

**Fig. 3.**
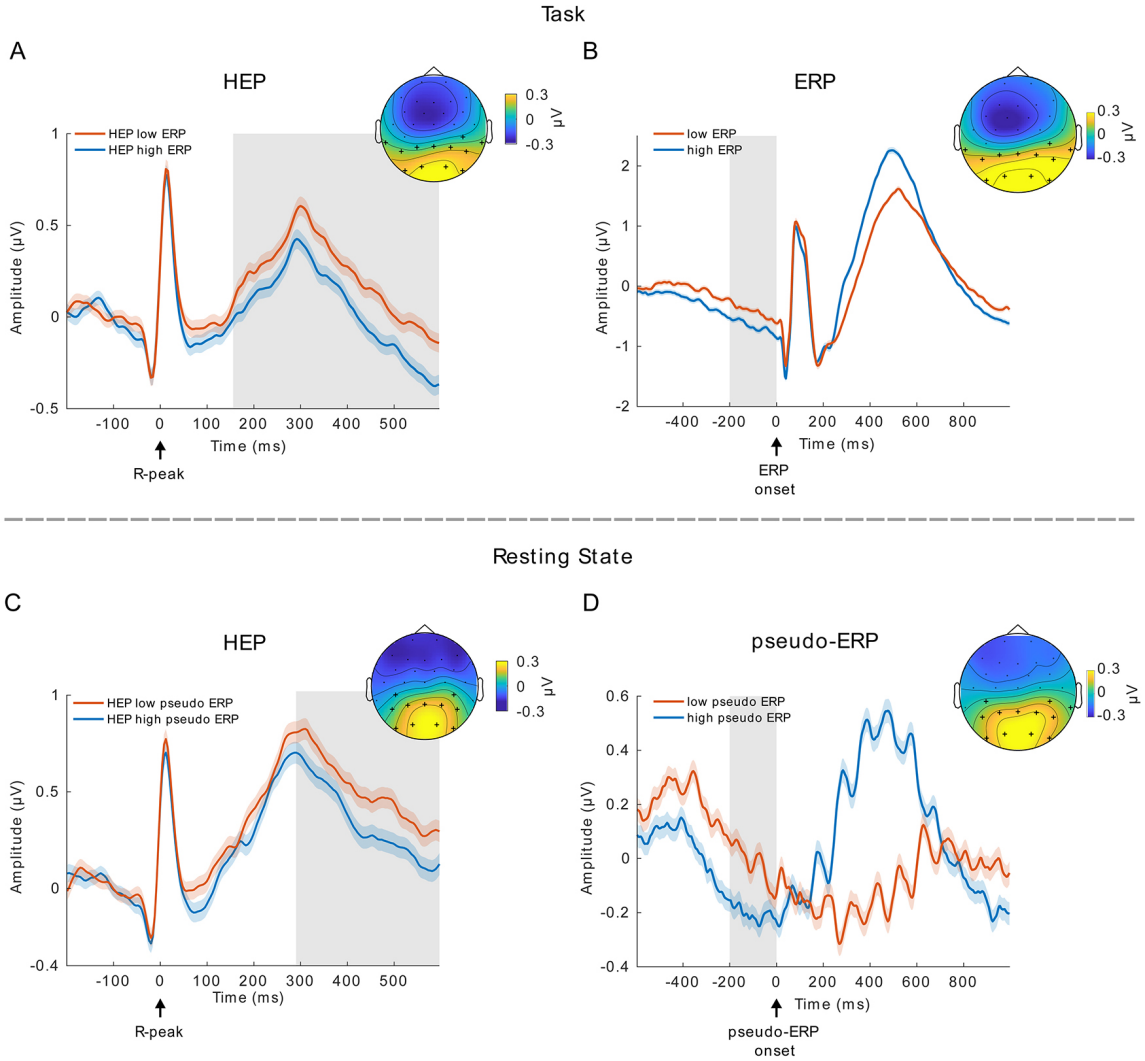
High-amplitude ERPs are preceded by low-amplitude HEPs—even in the absence of a task. (A) Pre-stimulus HEPs, categorized by the P300 amplitude of ERPs, reveal significantly higher HEP amplitudes preceding trials associated with low-amplitude P300 ERPs, particularly over posterior electrodes. (B) A comparison of pre-stimulus activity for low and high P300 amplitude ERPs, in the time window from -200 to 0 ms, also shows a significant difference. (C) HEPs are significantly different if they are sorted by the amplitude of “pseudo-ERPs.” (D) Resting-state pseudo-ERPs show a significant difference in the time window of -200–0 ms. Shaded regions around the lines represent ± Standard Error of the Mean (SEM), gray boxes:*p*≤ 0.001, topoplots are averaged over time of significant differences and black crosses indicate significant channels.

Given that HEP amplitudes were shown to be higher for interoceptive than for exteroceptive attention (Al et al., 2020,^2021^;García-Cordero et al., 2017;Petzschner et al., 2019;Villena-González et al., 2017) and P300 amplitudes index an attentional shift toward external stimuli (Kok, 2001;Polich, 2007), we expected to observe a negative relationship between HEP and P300 amplitudes. Since, on average, 27.2 target trials had a heartbeat in the pre-stimulus time of interest, the median split resulted in 13.6 trials per high and low P300 amplitude condition. In line with our prediction, we found significantly smaller HEPs preceding high amplitude P300 ERPs in the time between 156 and 600 ms in a posterior electrode cluster (Fig. 3A; cluster sum(*t*) = 2842,*p*≤ 0.001, significant channels: CP6, TP9, TP10, P3, P4, P7, P8, Pz, O1, O2, PO9, PO10). This was reversed for frontal electrodes, with larger HEPs for larger P300 ERPs in a similar time window (time range: 144–600 ms, cluster sum(*t*) = -4562,*p*≤ 0.001, significant channels: Fp1, Fp2, F3, F4, F7, F8, Fz, FC1, FC2, FC5, FC6, C3, C4, T7, Cz, CP5). The ECG was not significantly different between the high and low P300 conditions (temporal cluster-based permutation,*p*> 0.05), indicating that the effect is independent of cardiac field artifacts.

**Fig. 4.**
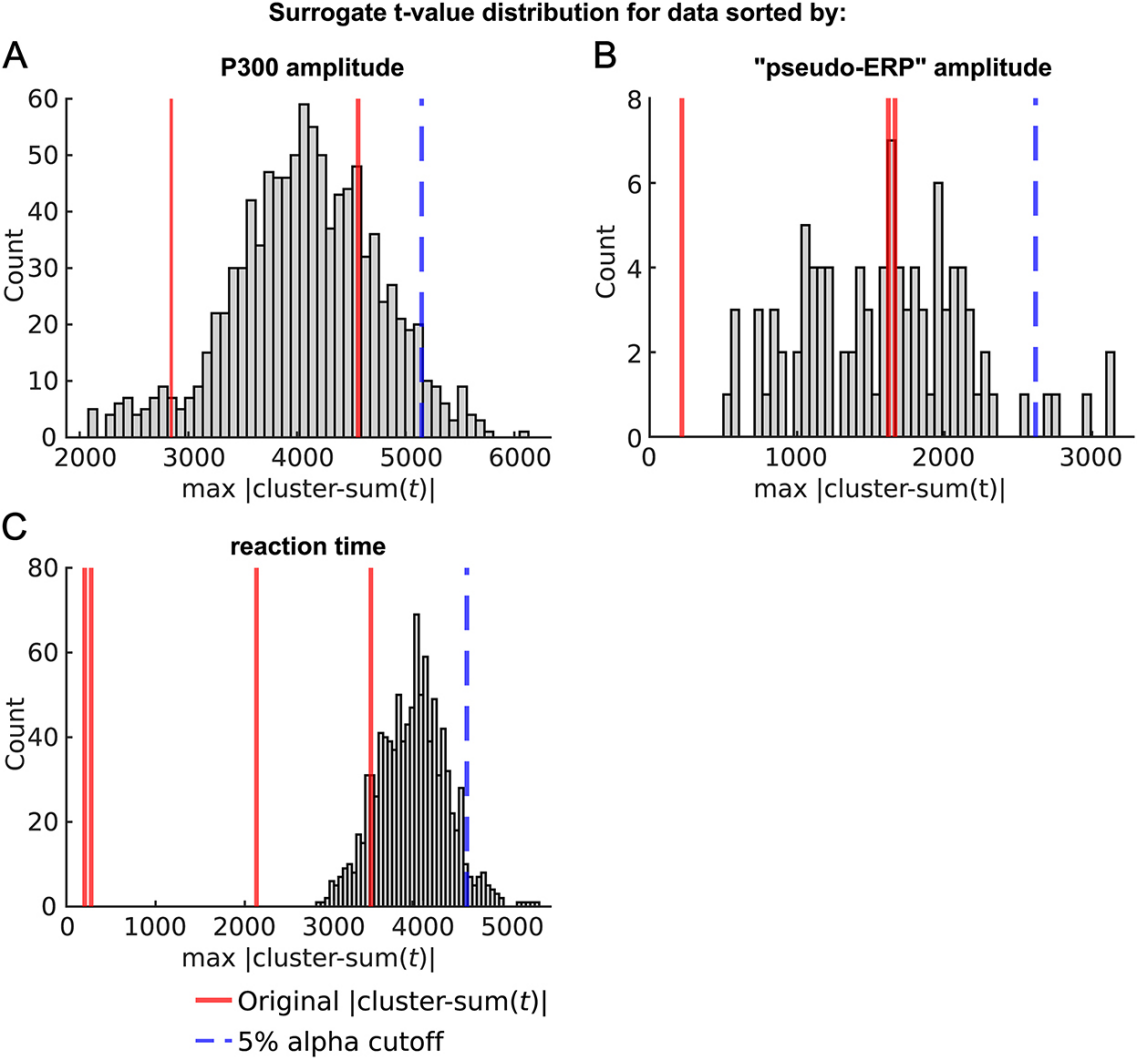
Distribution of maximum |cluster-sum(*t*)|-values obtained from surrogate heartbeat control analyses. Histograms illustrate |cluster-sum(*t)*|-values for surrogate heartbeat differences with shuffled R-peak onsets and original HEP effects for all significant clusters (red lines). The related trials are sorted in (A) high versus low P300 amplitude, (B) high versus low “pseudo ERP” amplitude, and (C) fast versus slow reaction time (RT). The dotted blue lines represent the 5% alpha cutoff.

However, when inspecting the sorted P300 ERPs, they show significant pre-stimulus baseline differences (Fig. 3B; average between -200 and 0 ms, cluster-based permutation in space, cluster sum(*t*) = 121.5,*p*≤ 0.001, significant channels: CP6, TP9, TP10, P3, P4, P7, P8, Pz, O1, O2, PO9, PO10). To further investigate whether this pre-stimulus difference can drive the observed HEP effect, we performed a surrogate heartbeat analysis. The original*t*-values for the positive and negative clusters were not significantly greater than the corresponding*t*-values obtained by the surrogate analysis at an alpha level of α = 0.05 (Monte-Carlo*p*-values positive cluster:*p*= 0.949, negative cluster:*p*= 0.763;Fig. 4A), indicating that the observed differences between HEPs in the high and low P300 condition are not time-locked to the R-peak.

### 3.2 During rest, HEP differences precede pseudo-ERPs with high and low amplitude in P300 time window

To determine whether the observed association between HEPs and P300 amplitudes was related to the data analysis approach rather than the auditory oddball task, we performed a control analysis using resting-state data. We copied the oddball task triggers from the task recording to resting-state data of the same subjects and repeated the same analyses. Since real HEPs are present during resting state but task-evoked activity is absent, this approach allowed us to investigate whether the HEP differences are truly task related or a result of the data-sorting process. We sorted the resting-state data into high- and low-amplitude conditions based on the 250–600 ms post-stimulus time window, thereby creating high- and low-amplitude “pseudo-ERPs” without any actual task-evoked activity (Fig. 3D). We then examined the HEPs occurring in the pre-stimulus time of these “pseudo-ERPs” (Fig. 3C). The resting-state control analysis included 1730 subjects, and data processing was performed identically to that of the real task data. Using spatio-temporal cluster-based permutation*t*-tests, we observed that HEP amplitudes were smaller before trials with high “pseudo-ERP” amplitude between 292 and 600 ms in a posterior electrode cluster (Fig. 3C; cluster sum(*t*) = 1618,*p*≤ 0.001, significant channels: CP5, CP6, P3, P4, P7, P8, Pz, O1, O2, PO9, PO10). Additionally, negative clusters were present in the time 288–600 ms in a frontal electrode cluster (cluster sum(*t*) = -1663,*p*≤ 0.001, significant channels: Fp1, Fp2, F3, F4, F7, F8, Fz, FC1, FC2, FC5, FC6, C3, T7, Cz, FT9, FT10) and a second negative cluster in the time range: 64–132 ms (sum(*t*) = -218,*p*= 0.014, significant channels: Fp1, Fp2, F3, F7, F8, FC5, FC6, C3, T7, FT9, FT10). Consistent with these findings, we again observed a significant difference in the pre-stimulus time -200–0 ms of the sorted resting-state “pseudo ERPs” (Fig. 3D; cluster sum(*t*) = -94,*p*≤ 0.001, significant channels: CP5, CP6, TP10, P3, P4, P7, P8, Pz, O1, O2, PO9, PO10). These results suggest that the HEP difference we observed when comparing high versus low real P300 trials (not “pseudo-ERPs”) during the task is in fact independent of the task. Instead, it likely represents an artifact stemming from the trial sorting process, possibly due to regression toward the mean (Bland & Altman, 1994). This conclusion is further supported by a surrogate heartbeat control analysis, which showed that the sum(*t*)-values of HEP differences during rest were not bigger than the sum(*t*)-values of the surrogate data (Fig. 4B; 100 permutations, positive cluster:*p*= 0.52, negative cluster 1*p*= 0.45, negative cluster 2*p*= 1). This indicates that the observed differences could not be attributed to a truly heartbeat-related effect.

### 3.3 HEP differences precede trials with fast and slow reaction times

**Fig. 5.**
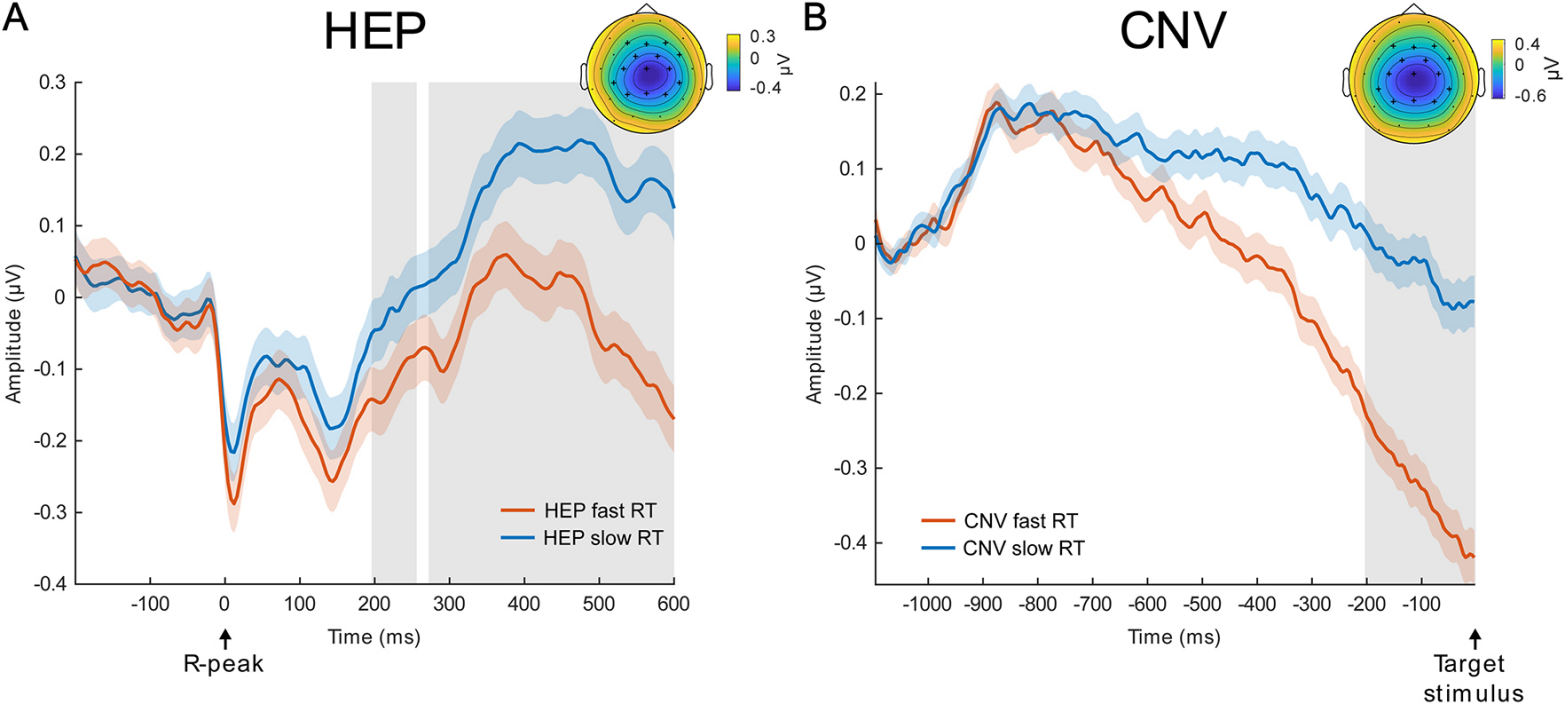
HEP and CNV differences before fast and slow reaction time trials. (A) HEP difference before fast and slow RT trials with significant differences in central electrodes. (B) Difference in contingent negative variation (CNV) before fast and slow reaction time (RT) trials. Pre-stimulus activity was compared between -200 and 0 ms and showed a significant difference. A baseline correction was applied based on the average amplitudes in the -1100 to -1000 ms time window. Gray area indicates*p*≤ 0.001, shades around the lines are ±SEM. Topoplots are averaged over time with the largest significant difference and significant channels are displayed as black cross.

The next aim of our study was to investigate whether HEP amplitudes relate to reaction times as a behavioral marker of task performance. Since reaction times relate to on-task attention (Posner & Boies, 1971;Yamashita et al., 2021), we hypothesized to see an inverse relationship with HEP amplitudes, with lower HEP amplitudes prior to faster reaction times. We reasoned that when attention is predominantly directed toward external target stimuli, interoceptive signals, including cardiac information reflected in the HEP, may receive fewer processing resources (Villena-González et al., 2017). In addition, by using reaction times as a behavioral measure, we aimed to circumvent the artifacts induced by sorting post-stimulus EEG data as shown in the previous sections. We median-split trials in fast and slow RT categories and compared HEPs in the respective pre-stimulus time. Average reaction times were 432 ms (*SD*= 110 ms) for the fast condition and 529 ms (*SD*= 130 ms) for the slow condition. Based on spatio-temporal cluster-based permutation*t*-tests, we observed smaller HEP amplitudes for faster RT trials in two central electrode clusters (Fig. 5A; negative cluster 1: 272–600 ms, cluster sum(*t*) = -3412,*p*≤ 0.001, significant electrodes: F3, F4, Fz, FC1, FC2, FC6, C3, C4, Cz, CP5, CP6, P3, P4, Pz; negative cluster 2: 196–256 ms, cluster sum(*t*) = -277,*p*= 0.002, significant electrodes: FC1, FC2, FC6, C4, Cz, CP6, P4, Pz; positive cluster 1: 368–600 ms, cluster sum(*t*) = 204,*p*≤ 0.001, significant electrodes: Fp1, Fp2, F7, F8, T7, T8, FT9, FT10, TP9, TP10, P7, O1, PO9, PO10; positive cluster 2: 320–356 ms, cluster sum(*t*) = 204,*p*= 0.019, significant electrodes: Fp1, Fp2, F7, F8, FC5, T7, FT9, FT10, TP9). The ECG was not significantly different between the fast versus slow RT conditions (temporal cluster-based permutation,*p*> 0.05).

However, upon closer inspection of the pre-stimulus time of the ERP trials split into a fast and slow RT category, it becomes apparent that the EEG activity before stimulus onset is already different between the two groups (Fig. 5B). Using spatial cluster-based permutation tests to compare the average amplitude in the -200–0 ms time, we found a significant difference between the conditions in a central electrode cluster (cluster sum(*t*) = -163,*p*≤ 0.001, significant electrodes: F3, F4, Fz, FC1, FC2, FC6, C3, C4, Cz, CP5, CP6, P3, P4, Pz) with more negative amplitude for faster RT trials. Since the potential is leading up to the target stimulus which requires the subjects to press a button, and its specific spatial distribution, we consider it to be a contingent negative variation (CNV,Walter et al., 1964). To investigate whether the pre-stimulus CNV difference between fast and slow RT trials is driving the HEP effects, we again performed a surrogate heartbeat analysis with permuted R-peak timings within each subject per condition. We created 1000 surrogate datasets with shuffled R-peak onsets. Comparing the original sum(*t*) values with the surrogate sum(*t*) values, we found that the effects were not related to the R-peak, with*p*-values of 0.878 and 1.00 for the negative clusters, and 0.336 and 1.00 for the positive clusters (Fig. 4C).

### 3.4 No residual pre-stimulus HEP differences after pseudotrial correction

**Fig. 6.**
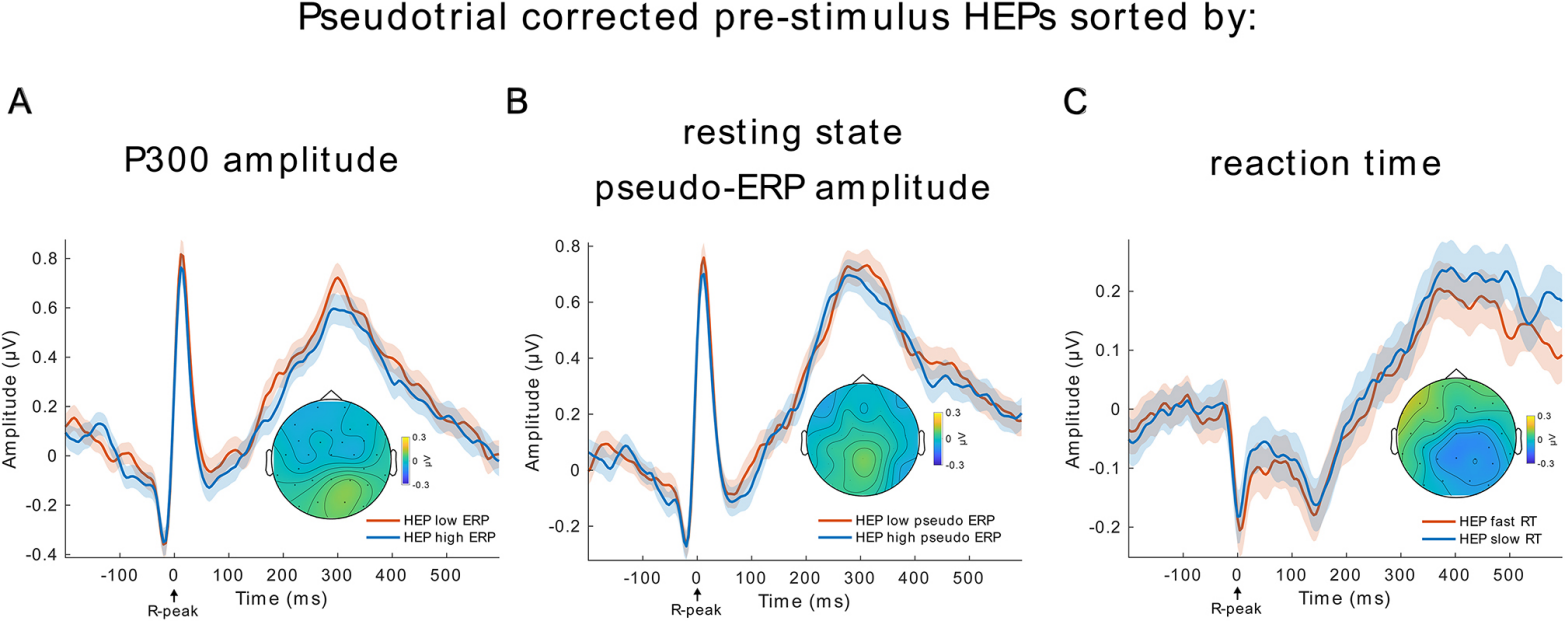
Pre-stimulus HEPs show no significant differences after pseudotrial correction. After pseudotrial correction, no significant differences can be found for pre-stimulus HEPs that were sorted based on (A) the amplitude of the subsequent ERP P300 amplitude during the oddball task, (B) the amplitude of pseudo-ERPs sorted in high versus low condition in the P300 time window during resting state, or (C) the reaction time speed during the oddball task. The average HEPs are based on the same electrode clusters used for the respective HEPs inFigures 3A, Cand5A. Shades around the lines are ±SEM.

In the prior analyses, we demonstrated that the differences in pre-stimulus HEP observed when categorizing trials into either high versus low P300 condition or a fast versus slow RT condition were, in part, heartbeat unrelated. However, we wanted to investigate whether genuine HEP differences might still exist while being masked by the spurious effects we observed (see alsoSection 3.5). Hence, we have adopted a pseudotrial correction method (Huang et al., 2017;Wainio-Theberge et al., 2021), which subtracts ongoing fluctuations from the HEPs and can thereby correct for slow potential differences. We repeated our cluster-based permutation tests for the pseudotrial corrected data and did not observe any significant condition differences for the pre-stimulus HEPs between high versus low P300 ERP amplitude, fast versus slow reaction times, or “pseudo-ERP” amplitude during resting state (Fig. 6; cluster-based permutation*t*-tests,*p*> 0.05). To further investigate whether the conditions could be considered statistically equivalent after pseudotrial correction, we conducted equivalence tests. For each participant, we averaged the corrected HEP data across the electrode and time cluster which was significant before pseudotrial correcting the data. The effect size of the difference between conditions was then compared with the smallest meaningful effect of a Hedge’s*g*= 0.19 (see methods for explanation of this boundary). After correction, the pre-stimulus HEPs across all conditions can be considered statistically equivalent, including those during the oddball task for high versus low P300 (*t*_1739_= -7.2,*p*< 0.01) and fast versus slow RT (*t*_1739_= 5.51,*p*< 0.01), as well as during resting-state high versus low pseudo-ERP (*t*_1730_= -7,*p*< 0.01). This suggests that the initially observed differences were likely driven by non-cardiac confounds rather than genuine HEP effects.

### 3.5 Removal of sorting-related artifacts by pseudotrial correction increases sensitivity to detect true HEP effects in simulated data

**Fig. 7.**
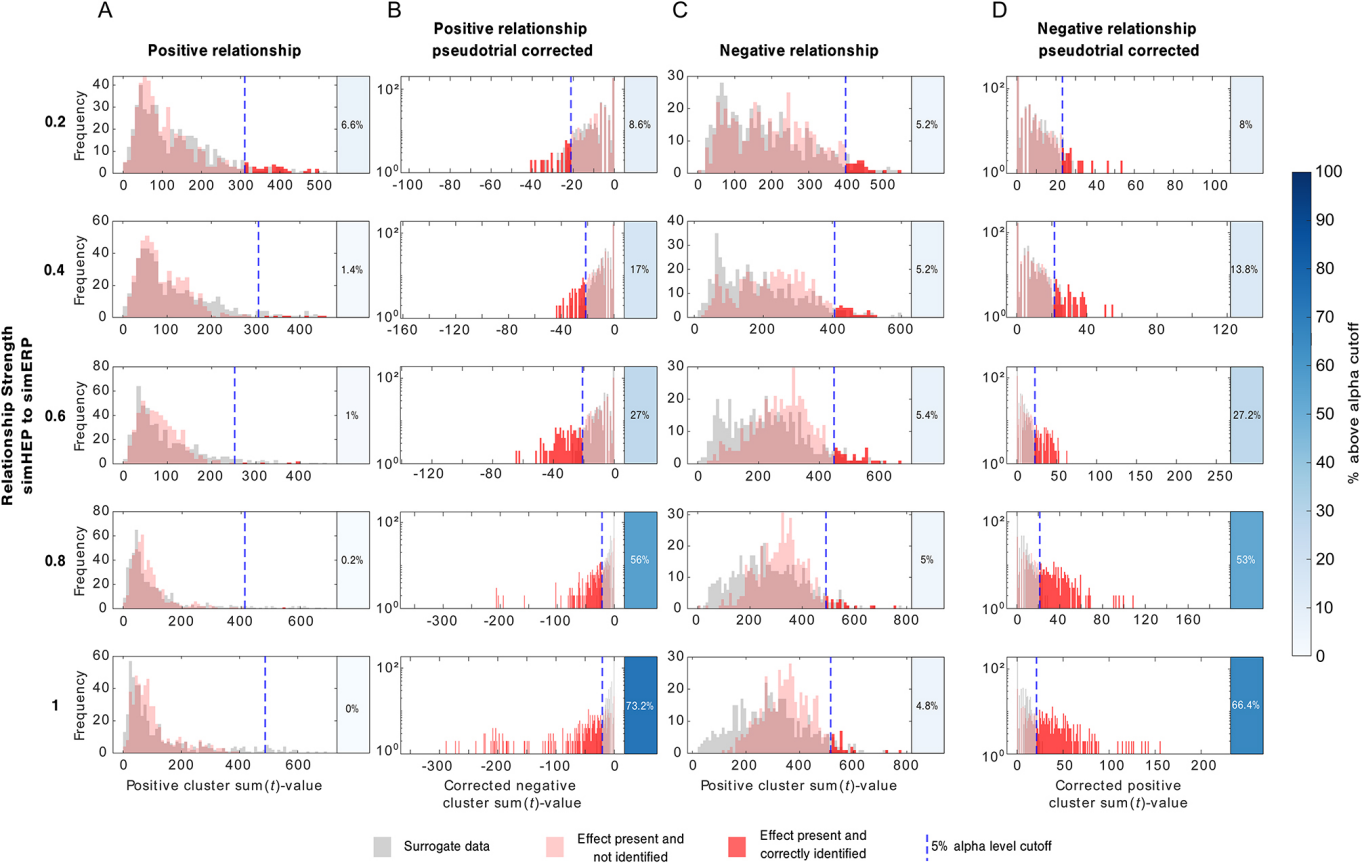
Pseudotrial correction and surrogate control analysis on simulated HEP to ERP relationships. Histograms visualizing cluster sum(*t*)-values obtained from 500 permutations of simulated data where an association between pre-stimulus simHEP and simERP was present (red) and corresponding surrogate data (gray). The dotted line indicates the 5% alpha level obtained from the surrogate data. Sum(*t*)-values greater than this cutoff are considered significant in a surrogate heartbeat control analysis (dark red). Boxes right next to the histograms indicate which percentage of the permutations would be considered significant, and hence reflects the power to detect an effect. Rows relate to different linear relationship strengths between pre-stimulus HEP and post-stimulus-evoked response. Columns reflect positive or negative relationship between pre-stimulus HEP and post-stimulus-evoked response (A & C) and their pseudotrial corrected counterparts (B & D). The y-axis of histograms after pseudotrial correction (B, D) is log-scaled for better visibility.

Differences in HEPs between conditions can be spuriously introduced by overlap with ongoing brain activity (Park & Blanke, 2019). To account for this, surrogate heartbeat control analyses have been proposed. However, when the surrogate heartbeat procedure is used to control for the influence of non-heartbeat-related activity, true HEP effects may be missed if they are mixed with confounds. Hence, we have adopted a pseudotrial correction method (Huang et al., 2017;Wainio-Theberge et al., 2021), which subtracts confounding factors from the HEPs. To demonstrate that pseudotrial correction can indeed uncover genuine HEP effects if they are mixed with other processes, we simulated EEG data where task-evoked activity (simERP) relates to pre-stimulus HEP amplitudes (simHEP) in positive and negative direction (see MethodsSection 2.12for details). The simulated data were sorted based on the 250–600 ms post-stimulus time window (corresponding to the P300 time range). Splitting data into high- and low-amplitude conditions can have long-lasting effects on the EEG, which can reach the pre-stimulus time (seeFig. 3B, D) and induce spurious pre-stimulus HEP effects (see Resultssection 3.1&3.2). To investigate whether true simHEP effects can be identified in the presence of sorting-induced artifacts, we performed a surrogate heartbeat control analysis on these data. The same analysis was repeated 500 times, while for each iteration the sum(*t*)-value of the largest cluster was retained for both: data where a relationship was present between simHEP and simERP (original data) but also their corresponding surrogate data. Based on the surrogate data, we defined a 5% alpha cutoff. We then calculated the percentage of permutations where the cluster sum(*t*) of the original data was larger than this cutoff. We found that significant HEP effects were detected in 0–6.6% of cases, demonstrating the effectiveness of surrogate heartbeat analysis in controlling for false-positive findings in the presence of heartbeat-independent confounds (type I error) (Fig. 7, column A, C). However, even with a correlation of 1 between simHEP and simERP, genuine HEP effects were found in only 0% (positive relationship) or 4.8% (negative relationship) of the cases. Since real effects are present, but remain undetected (type II error), we performed a pseudotrial correction on the same data. We show that the correction can remove some of the spurious differences and uncover genuine simHEP effects, as the power to detect differences increased up to 73.2% (Fig. 7B, positive relationship) and 66.4% (Fig. 7D, negative relationship). It is important to note that the effects were simulated with a conservatively small SNR of 3.3 dB for simERPs and -9.3 dB for simHEPs, which influences the ability to detect present effects.

### 3.6 HEP differences between oddball task and resting state

As shown in the Resultssection 3.3, the pre-stimulus heartbeat-evoked potentials (HEPs) in our task overlapped with a slow stimulus-preceding potential known as the contingent negative variation (CNV). Also, we demonstrated that these CNVs can be effectively removed from HEPs using pseudotrial correction. In simulations, we could show that slow artifacts can dominate HEP effects, which prevents other potentially present HEP effects from being found. Hence, our next aim was to investigate the relationship between resting-state and task HEPs before and after pseudotrial correction. We hypothesized that, due to the overlap with the CNV, HEPs would mirror its spatio-temporal distribution and appear lower in central electrodes during task than during rest prior pseudotrial correction. Furthermore, we anticipated that pseudotrial correction would remove the spurious effect and uncover differences in HEPs between rest and task that are invisible before correction.

**Fig. 8.**
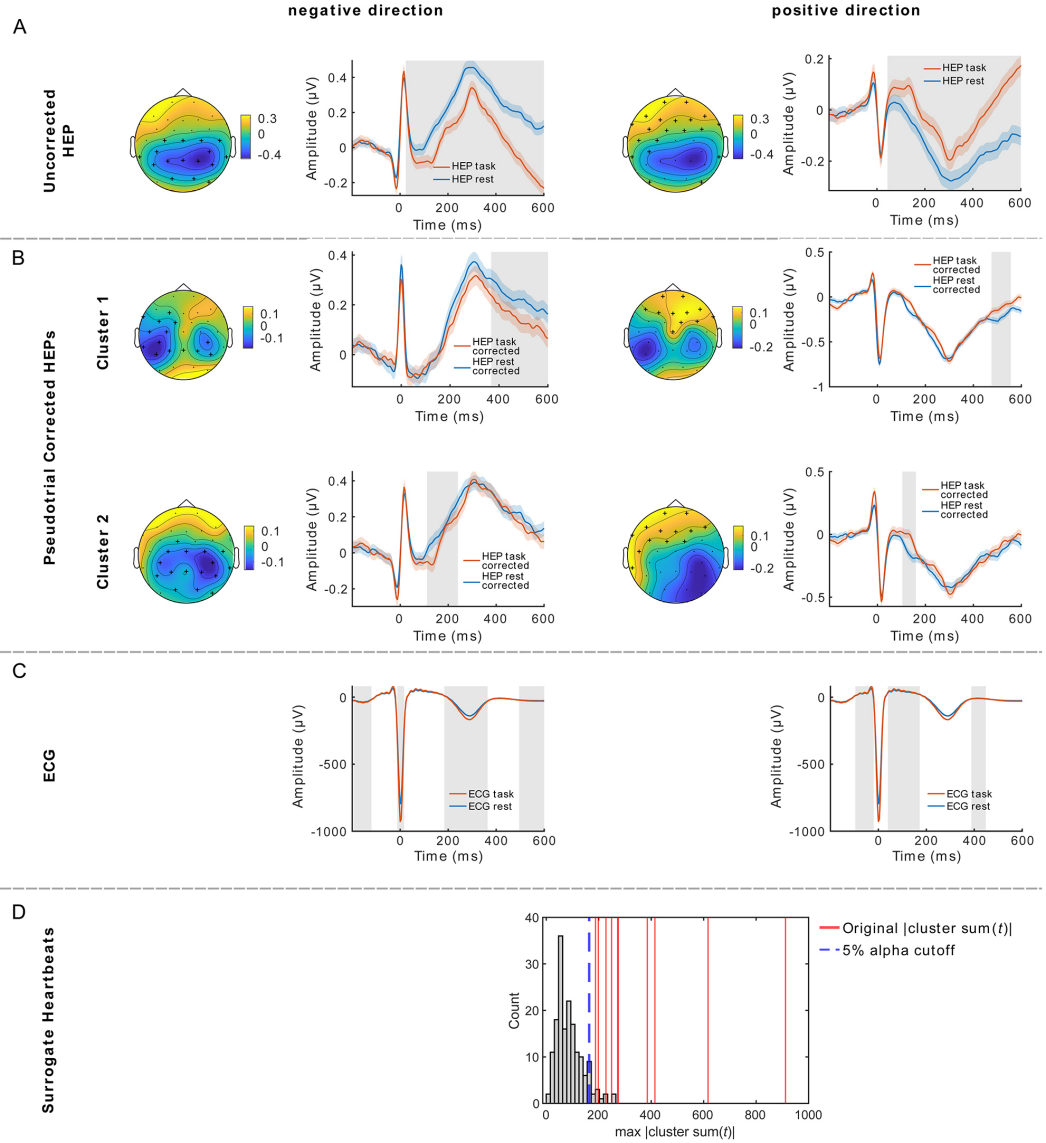
HEP differences between Oddball task and resting state. Differences in HEPs between Oddball task and resting state in positive and negative directions (A) before and (B) after pseudotrial correction. After correction, several clusters were found and the largest first two are illustrated. (C) ECG differences between task and rest are illustrated. Shades around the lines are ±SEM, gray boxes:*p*≤ 0.05, Topoplots are averaged over time of significant differences and black crosses highlight significant channels. (D) Histograms visualizing the surrogate heartbeat control analysis. All clusters of the original data have larger absolute cluster sum(*t*)-values (red lines) than the 5% alpha cutoff (blue dashed lines) obtained from the maximum surrogate statistics (gray bars).

Before pseudotrial correction, we only observe two clusters in positive as well as negative direction (Fig. 8A; positive cluster 1: 44–600 ms, cluster sum(*t*) = 8046,*p*≤ 0.001, positive cluster 2: -72–8 ms, cluster sum(*t*) = 431,*p*≤ 0.001; negative cluster 1: 24–600 ms, cluster sum(*t*): -8906,*p*≤ 0.001; negative cluster 2: -72–20 ms, cluster sum(*t*): -375,*p*= 0.002). Surrogate heartbeat control analysis (100 iterations) indicates that these clusters are heartbeat unrelated, as all maximum |sum(*t*)| values obtained from shuffled data are larger than the original effects.

Importantly, the heartbeat-independent effects were removed after pseudotrial correction, revealing several clusters distributed in time and space. The first two clusters with the largest sum(*t*) values are illustrated inFigure 8B(for a summary of all clusters, seeSupplementary Table S2). Surrogate heartbeat control analysis indicates that all clusters have |sum(*t*)| values larger than 95% of the maximum surrogate statistic obtained from shuffled data. Therefore, we can conclude that the effects we observe after pseudotrial correction reflect differences in activity which are time-locked to the heartbeat.

**Fig. 9.**
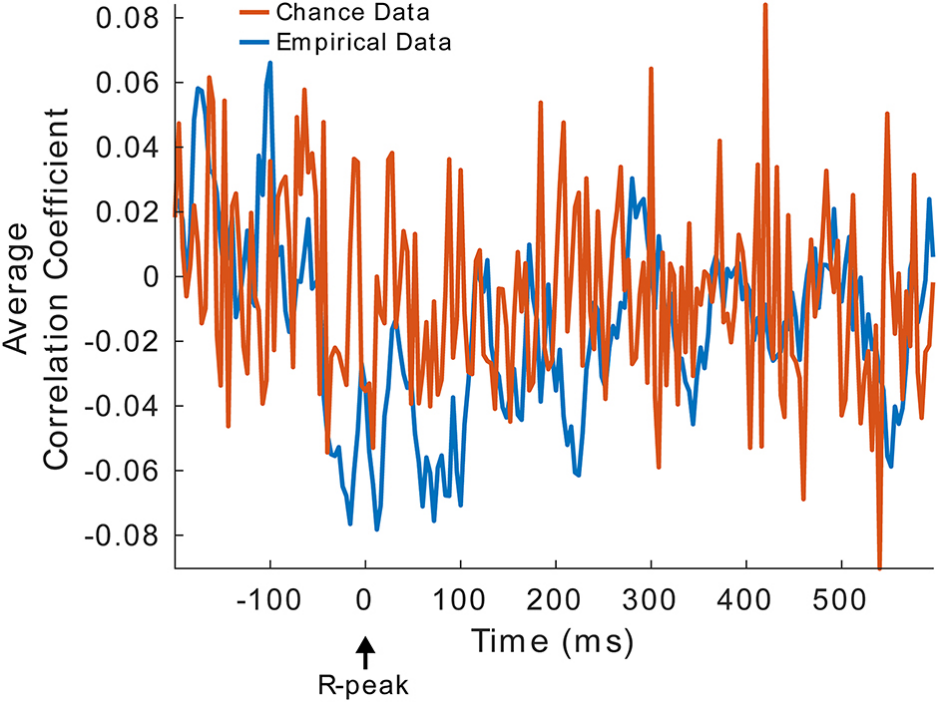
No Significant Correlation betweenΔECGandΔEEGin time range of HEP analysis. Correlation between resting-state and task-related differences in ECG (ΔECG) and EEG (ΔEEG) across all channels and time points and corresponding chance data obtained by correlation ofΔEEGwith randomizedΔECGdata. No clusters of significant differences were found (*p*> 0.05). Time courses for illustration are obtained from the Cz electrode and grand averaged across subjects.

To assess the potential influence of cardiac field artifacts on these differences, we compared the ECG between resting state and task by a temporal cluster-based permutation*t*-test. Several significant differences could be observed (Fig. 8C; largest cluster in positive: cluster sum(t): 303,*p*≤ 0.001, time: 4–172 ms, and negative direction: cluster sum(t): -849,*p*≤ 0.001, time: 184–364 ms, all other clusters*p*≤ 0.001), with a maximum effect size of a Cohen’s*d*-0.87 at 276 ms, coinciding with the T-wave (Supplementary Fig. S7; obtained using fieldtrips ft_statfun_cohensd,Oostenveld et al., 2011), indicating possible CFA-induced HEP differences when comparing resting state and task. Additionally, significant differences in heart rate were present, with 61.3 (*SD*= 8.1) beats per minute (bpm) during rest and 63.7 (*SD*= 8.2) bpm during the task (paired*t*-test,*p*= 1.0828e-39, effect size of the difference Cohen’s*d*= 0.29). To further investigate whether CFA could explain the HEP differences, we tested whether the difference between resting state and task ECG (ΔECG)correlates with the difference between resting state and task EEG (ΔEEG). Yet no significant clusters were found in the HEP time window of interest (Fig. 9), suggesting that CFA alone does not account for the observed HEP differences.

## 4 Discussion

Heartbeat-evoked potentials, which reflect the cortical processing of afferent signals generated by the heartbeat, have been shown to relate to different cognitive, emotional, or pathophysiological conditions (Engelen et al., 2023;Park & Blanke, 2019). However, when studying HEPs across different contexts, it is crucial to control for potential overlaps with ongoing neural activity unrelated to the heartbeat (Azzalini, et al., 2019). In the present study, we aimed to highlight the pitfalls of uncontrolled analyses in heartbeat-evoked potential (HEP) research and demonstrate how appropriate control analyses, such as pseudotrial correction (Wainio-Theberge et al., 2021) and surrogate heartbeat analysis (Babo-Rebelo, Richter, et al., 2016;Park & Blanke, 2019), can effectively account for heartbeat-unrelated confounds. We applied these techniques in an analysis of pre-stimulus HEPs during an auditory oddball task where HEP effects can be spuriously introduced due to differences in underlying EEG activity unrelated to the heartbeat. Furthermore, using both simulated and real data, we show that pseudotrial correction has the potential to remove these confounding activities and uncover genuine HEP effects.

### 4.1 Pre-stimulus HEPs effects can be spuriously induced by underlying differences in neural activity

It was previously proposed that HEP amplitudes may serve as a marker for attentional processes—with higher amplitudes being indicative of interoceptive attention, and lower amplitudes reflecting exteroceptive attention (Al, et al., 2020;Petzschner et al., 2019). Given that P300 ERP is a signature of task-relevant attentional processes (Herzog et al., 2021;Kok, 2001;Polich, 2007), we expected P300 amplitudes to be inversely related to pre-stimulus HEP amplitudes. To test this hypothesis, we sorted single trial P300 ERPs into high- and low-amplitude subgroups and investigated whether there is a significant difference between the corresponding pre-stimulus HEPs. Initially, we indeed find the expected significant differences. However, when performing the same analysis on resting-state data, generating high and low “pseudo ERP” conditions, we observe a similar pattern. This is surprising because, if task-relevant cognitive processes influence HEPs, such differences should not appear during rest when no task is performed.

To investigate the origin of the observed HEP differences further, we first tested whether the effects we find are actually related to the heartbeat. We created surrogate heartbeats by shuffling the original HEP onset timings multiple times and repeated the same statistical analysis on these surrogate HEPs to generate a null distribution of effects. By comparing our original results with this surrogate null distribution, we found that the initial HEP differences could not be considered significantly associated with the heartbeat. This suggests that the initially observed HEP effects were likely unrelated to the heartbeat.

Thus, the question arises why we initially observed differences in pre-stimulus HEPs? One potential explanation lies in the inherent temporal dependencies of the EEG signal, where the up- and down-states of the signal do not fluctuate randomly, but are organized in a sequence over time. Due to the cyclic nature of the EEG signal, when investigating the association between high amplitudes in the post-stimulus time (up state), it is likely that they are preceded by low amplitudes in the pre-stimulus time (down state) and vice versa. This can create an inverse correlation between the two time points. Due to the relatively long time range spanning pre- and post-stimulus time in our experiment, especially slower fluctuations will shape the relationship between the two. These fluctuations are characterized by oscillations in the low frequency <4 Hz range (e.g., Delta rhythm and below), but also faster oscillations can influence their dynamics on longer time-scales via a baseline shift mechanism (Studenova et al., 2023) or through phase amplitude coupling (Tort et al., 2010). Additionally, regression to the mean may contribute to the observed inverse relationship. When selecting trials based on extreme values (high or low P300 amplitudes), subsequent measurements of pre-stimulus HEPs are statistically likely to be closer to the average, creating an artificial inverse correlation (Bland & Altman, 1994;Tu & Gilthorpe, 2007).

To avoid the pitfalls of post-stimulus data sorting, we next used reaction time (RT) as a behavioral marker of task engagement and compared pre-stimulus HEPs in fast versus slow RT trials. We hypothesized that pre-stimulus HEPs would be related to reaction times, since reaction time speed is related to on-task attention (Posner & Boies, 1971), and HEPs are lower if the focus of attention is on task-related processes (Al et al., 2020;Petzschner et al., 2019;Zaccaro et al., 2022). Indeed, we initially observed lower HEPs in fast RT trials. However, as with the P300- or resting-state sorting, our surrogate heartbeat analysis indicated that this effect was not truly heartbeat-locked. One reason for this effect was a slow preparatory potential linked to reaction times in the pre-stimulus time of target stimuli. The potential likely reflects contingent negative variation (CNV), associated with anticipatory attention and response preparation (Hillyard, 1969;Walter et al., 1964). The topography of the initially significant HEP differences closely resembles that of CNV, suggesting that sorting by RT inadvertently confounds HEP with CNV activity.

Nonetheless, even though the HEP differences we observe all seem heartbeat unrelated, the possibility still exists that genuine HEP effects are present, but are not found because they are masked by the confounds. We addressed this by performing a pseudotrial subtraction which effectively removes heartbeat-unrelated activity from the HEPs. After applying this correction, the HEP differences for all conditions (HEPs sorted by: P300 amplitude, “pseudo ERP” amplitude, RT) were no longer significant, indicating they were attributable to non-cardiac-related processes.

For the P300 amplitude sorted HEPs, our results are in contrast to previously reported inverse relationships between pre-stimulus HEPs and P300 amplitudes (Al et al., 2020,^2021^;Marshall et al., 2019). While differences in the used tasks (e.g., somatosensory detection or reward incentive paradigm) and analysis strategies are apparent, further research is needed to clarify the scenarios in which a relationship between pre-stimulus HEPs and the P300 ERP can be validated. In case of RT sorted HEPs, our observation that spurious CNV-related effects can be removed from HEPs by pseudotrial subtraction is in line with recent work (Aprile et al., 2024), showing no association between HEP amplitudes and anticipatory attention after pseudotrial correction. Consistent with this,Marshall et al. (2019)also observed no direct association between pre-stimulus HEP and CNV amplitude or reaction times. Consequently, our results, therefore, provide no evidence for a direct interaction between reaction times and pre-stimulus HEP amplitudes.

### 4.2 The potential of pseudotrial correction to uncover genuine HEP effects

Although our task-based analyses did not reveal significant HEP effects after controlling for confounding factors, we aimed to demonstrate the potential of pseudotrial correction to uncover subtle effects that might be obscured by overlapping heartbeat-unrelated activity. To achieve this, we simulated data where pre-stimulus HEPs (simHEP) are related to post-stimulus ERPs (simERP) in both positive and negative directions. As in our previous analysis, we sorted the simERPs into different amplitude conditions, and investigated whether pre-stimulus simHEPs would differ significantly between these groups.

Before applying pseudotrial correction, most of the simHEP differences would be considered heartbeat independent based on a surrogate heartbeat analysis. However, after applying pseudotrial correction, the likelihood to correctly identify genuine simHEP effects increases with the strength of the relationship between simulated simHEP and simERP. This finding demonstrates that pseudotrial correction effectively reduces the influence of artifacts and allows for a more accurate identification of true HEP effects.

### 4.3 Pseudotrial correction can uncover previously undetected HEP effects in real data

To further show the potential of pseudotrial correction to remove spurious activity in real (not simulated) data, we compared HEPs obtained from resting-state data with those from the pre-stimulus period of target stimuli from the oddball task. Since HEP amplitudes are higher when attention is focused on internal stimuli (Al et al., 2020;García-Cordero et al., 2017;Marshall et al., 2017;Petzschner et al., 2019), we expected higher amplitudes during rest compared with the task condition (Al et al., 2021). In our task-based analyses, we observed that pre-stimulus HEPs overlapped with the contingent negative variation (CNV), whereas this overlap should not occur during resting state. Because the CNV is characterized by a downward drift on which the HEPs are superimposed, we anticipated that HEP amplitudes would appear spuriously lower during task as compared with rest. This was indeed the case, as supported by a surrogate heartbeat control analysis, indicating that much of the observed task-versus-rest HEP difference was unrelated to the heartbeat. Notably, after applying pseudotrial correction to the HEPs, the initially strong differences disappeared, revealing multiple smaller clusters with varying temporal and spatial distributions. These clusters remained significant, even after application of surrogate control analysis, suggesting that they are likely heartbeat related. This indicates that pseudotrial correction successfully removed the CNV-related confound and uncovered heartbeat-locked effects.

Although surrogate heartbeat analysis and pseudotrial correction control for heartbeat-unrelated confounds, artifacts time-locked to the heartbeat can still influence the results. To investigate whether cardiac field artifacts (CFA;Dirlich et al., 1997), which represent the influence of the heart’s electrical activity on EEG signals, could affect the HEP results, we compared the ECG between resting-state and task conditions. Indeed, we observe several clusters of significant differences in the ECG between resting state and task, with a large effect of 0.87 coinciding with the T-wave. These are potentially driven by the difference in heart rate between rest and task. To assess whether the HEP differences between rest and task were driven by the CFA, we performed an additional control analysis. We first computed the differences between rest and task ECG (ΔECG) and between rest and task EEG (ΔEEG). Then we performed a cluster-based correlation analysis across all time points and channels betweenΔECGandΔEEG. Since no cluster of significant correlations within the time range of our HEP analysis was found (-200 to 600 ms around the R-peak), the analysis suggests that the observed HEP effects are not attributable to CFA only.

Overall, our analysis highlights the potential of pseudotrial correction to uncover otherwise hidden effects. Importantly, we demonstrate that surrogate heartbeat control analysis can be applied to pseudotrial corrected data to account for residual noise or condition differences introduced by the pseudotrial subtraction. Therefore, by combining both methods, an effective control for heartbeat-independent type I and II errors is achieved.

### 4.4 Implications for future research on HEPs

The theoretical implications of slow potential differences in the ongoing neural activity on HEP amplitudes have been highlighted in prior work (Azzalini, et al., 2019;Park & Blanke, 2019). Importantly, many studies already incorporate surrogate heartbeat analyses to control for potential false positive findings (e.g., a non-exhaustive listAzzalini et al., 2021;Babo-Rebelo et al., 2016;Marshall et al., 2017,^2022^;Park et al., 2014;Perogamvros et al., 2019;Simor et al., 2021). Our study demonstrates that, while this method effectively controls for false positives related to non-heartbeat confounds, genuine HEP effects can be missed if confounds are present. Therefore, combining pseudotrial correction and surrogate heartbeat analyses offers a solution to the problem of overlapping activity. For instance,Tanaka et al. (2023)report that their observed HEP effects may be a result of the overlap between task-evoked activity and their studied HEPs. Here, application of the correction methods could help to delineate the true task effect on HEPs.

Another important consideration in HEP research is whether and how to apply baseline correction. As previously suggested, due to the cyclic nature of the heartbeat, baseline correction can be problematic as the baseline window is not necessarily free of activity originating from the previous heartbeat (Azzalini, et al., 2019;Banellis & Cruse, 2020;Kern et al., 2013;Park et al., 2014;Petzschner et al., 2019). To address this concern, we performed all our main analyses with and without applying baseline correction. In our study, the observation of no pre-stimulus HEP effects in relation to P300 or reaction time was consistent regardless of baseline correction (seeSupplementary Figs. S1–S4). However, some observations should be noted. First, without baseline correction, we observed significant differences in the baseline interval in RT-sorted HEPs (seeSupplementary Materials S2;Supplementary Fig. 2A), raising the possibility that subtracting such a biased baseline could artificially alter HEP amplitudes. Importantly, pseudotrial correction removed this baseline difference (Supplementary Fig. 4C), illustrating its potential to serve as an alternative for baseline correction. By estimating and removing heartbeat-independent signals from the HEP, it ensures that remaining activity is genuinely linked to the heartbeat. This approach is especially useful when a completely “clean” baseline window is difficult to establish (e.g., when overlap with task-evoked activity is present), helping to prevent spurious findings. This example illustrates how baseline correction can substantially affect the detection of genuine heartbeat-related effects, warranting the need for careful evaluation of the baseline window if baseline correction is performed (Luck, 2014).

### 4.5 Limitations

First, the population we used consisted of an elderly sample aged between 40 and 80 years old. Since previous research has shown that resting-state HEPs are affected by aging (Kamp et al., 2021), it could be that our null-findings are specific to this age group. However, others have specifically examined potential interactions between young and old age groups with HEPs during different cognitive tasks and observed no effects of age (Aprile et al., 2024).

Our comparison of task versus rest HEPs illustrates the potential of pseudotrial correction to uncover effects that may remain undetected in uncorrected data. However, in our experiment, also differences in ECG and heart rate were present which can potentially lead to spurious HEP effects. Although our correlation control analysis did not indicate a strong influence of the CFA on HEP results, some limitations should be acknowledged. We only have a single ECG channel available, and previous studies have shown that condition-induced ECG changes can vary depending on ECG electrode placement (Gray et al., 2007). It is still possible that the changes in HEP we observe are a reflection of the CFA, but that EEG and ECG electrodes capture different orientations of this artifact. Understanding how the CFA translates to EEG signals is crucial and should be addressed in future HEPs studies, ideally by using multiple ECG electrodes to provide a broader coverage of the cardiac artifacts. Therefore, it is essential that our results are replicated in carefully controlled experiments where cardiac and other physiological parameters are kept constant across conditions. Similarly, an additional class of artifacts is based on movement of channels or tissue due to pulsating blood vessels (Kern et al., 2013). Similar to the CFA, some of the pulse-related variability might be removed after exclusion of heart-related ICA components as shown in simultaneous EEG-fMRI studies (Salimi-Khorshidi et al., 2014;Srivastava et al., 2005). However, it would be important to experimentally control for changes in pulse and associated pulse-related artifacts to ensure that the measured HEPs truly are of neuronal origin.

Importantly, neither surrogate heartbeat control nor pseudotrial correction can account for artifacts that are inherently time-locked to the heartbeat. These include cardiac field, stroke volume, and pulse-related artifacts, as well as other physiological or neural processes coupled with the cardiac cycle, such as microsaccades (Ohl et al., 2016). To ensure accurate interpretation of HEP effects, these factors should ideally be controlled through careful experimental design and complemented by data cleaning approaches (e.g., using ICA;Buot et al., 2021).

The surrogate heartbeat procedure generates a distribution of surrogate test statistics, which is compared with data obtained from real heartbeats. Since the resulting distribution is essential to make reliable comparisons, it has to be performed many times to serve as an effective control. However, the optimal number of iterations is not yet empirically established, and researchers choose a number between 100 (Babo-Rebelo, Richter, et al., 2016;Candia-Rivera et al., 2023;Engelen et al., 2022;Perogamvros et al., 2019), 200 (Park et al., 2016), 400 (Marshall et al., 2017), 500 (Azzalini, et al., 2019), and 1000 (Babo-Rebelo et al., 2019;Babo-Rebelo, Wolpert, et al., 2016) iterations. In our study, we selected different numbers of permutations for different analyses, primarily because the maximum number of feasible iterations is limited by available computational power, since performing spatio-temporal cluster-based permutation tests many times is computationally intensive. However, if effects are close to the significance threshold, it is advisable to increase the number of permutations to get a more accurate*p*-value estimate (Good, 1994).

With an average of 27 pre-stimulus heartbeats being included in the analysis, the number of epochs in our study is on the lower side compared with other studies (70–1600 epochs;Coll et al., 2021). This could result in higher within-subject variability and less robust results. While these effects are probably in part balanced out through the large sample size included in our analysis (1739 subjects), the results should ideally be replicated in analyses including more trials per participant. Furthermore, the large sample size also comes at the cost of detecting statistically significant differences that may not be meaningful (e.g., the relatively small difference in heart rate between rest and task with a Cohen’s*d*of 0.29 might not substantially affect HEPs but still achieves significance potentially due to the large sample size (Lantz, 2013)).

Another methodological limitation involves the use of a median split for dichotomizing continuous variables (P300 amplitudes or reaction times) into artificial groups which is known to reduce the power of statistical tests (MacCallum et al., 2002). However, even if alternative analyses approaches are used (e.g., regression-based approaches), the core challenge of discerning HEPs from slow, overlapping activity would remain, albeit in a less immediately visible form.

Lastly, regression-based ERP analyses are becoming more popular and offer a solution to the problem of overlapping ERPs (Burns et al., 2013;Ehinger & Dimigen, 2019;Smith & Kutas, 2015). In theory, pseudotrials can be added to the regression models as confounders to allow for the correction of heartbeat-unrelated activity. This form of pseudotrial correction needs to be established in future studies.

### 4.6 Conclusion

Our study highlights the issue that neural activities which are not triggered by the heartbeat can interfere with the HEP if they occur at the same time. While many studies investigate HEPs during tasks, it is essential to distinguish HEP from other task-related activations, such as stimulus-evoked responses or response preparation. In such experimental designs, even if careful statistical analyses are applied that incorporate different sources of confounders (e.g., cardiac and other physiological parameters), HEPs can still be confounded by heartbeat unrelated factors. We believe that careful experimental design, which avoids the overlap between heartbeats and task-related activities, in addition to adequate control analyses should be taken into account when investigating HEPs.

In this study we highlight the use of two control analyses which, when combined, represent a methodological advancement in the analysis of HEPs. By effectively isolating genuine HEP effects from confounding activity, these methods allow for more accurate interpretations of the interplay between neural processing of cardiac information and external stimuli.

## Supplementary Material

Supplementary Material

## Data Availability

Anonymized data can be requested by application at the LIFE-Study Department (https://ldp.life.uni-leipzig.de/). The code used for the analyses will be made available athttps://github.com/PaulSteinfathupon publication of the manuscript.
