# Supplementary material for: Validating genuine changes in heartbeat-evoked potentials using pseudotrials and surrogate procedures
Source: Imaging Neurosci (Camb). 2025 Jun 10;3:IMAG.a.30. doi: 10.1162/IMAG.a.30 (PMC12319741; doi:10.1162/IMAG.a.30)
Supplement: Supplementary Material [file imag.a.30_supp.pdf]

Supplementary Materials

Due to the cyclic nature of the heartbeat and the potential overlap of the baseline correction window with the previous heartbeat, we chose to repeat all our main analyses without performing a baseline correction.

S1 HEP differences precede ERPs with high and low P300 and "pseudo-ERP" amplitude

In line with the results obtained from baseline-corrected data, we observe significantly different HEPs before trials sorted into high and low P300 amplitude conditions during an auditory oddball task. Several significant clusters can be observed: positive cluster 1: sum(t): 2930, $p \leq 0.001$, time: 156–600 ms, channels:T8, FT10, CP6, TP9, TP10, P3, P4, P7, P8, Pz, O1, O2, PO9, PO10; negative cluster 1: sum(t): -4536, $p \leq 0.001$, time: 140–600 ms, channels: Fp1, Fp2, F3, F4, F7, F8, Fz, FC1, FC2, FC5, FC6, C3, C4, T7, Cz, FT9, CP5, negative cluster 2: sum(t): -345, $p \leq 0.001$, time: 28–132 ms, channels: Fp1, F3, F7, Fz, FC1, FC2, FC5, C3, Cz, CP5. The largest positive cluster is visualized in Supplementary figure 1A. Both clusters reflect a heartbeat independent process according to a surrogate heartbeat analysis (SFig. 3A, positive-cluster: $p = 1$, negative-cluster: $p = 0.1$).

Furthermore, also if the same analysis is repeated after copying task triggers to resting state data a similar picture emerges with several significantly different clusters: positive cluster 1: sum(t): 2691, p $\leq$ 0.001, 176–600 ms, channels: CP5, CP6, P3, P4, P7, P8, Pz, O1, O2, PO9, PO10; positive cluster 2: sum(t): 384, time: 56–152 ms, ,p $\leq$ 0.001; channels: CP6, P3, P4, P7, P8, Pz, O1, O2; negative cluster 1: sum(t): -3005, p $\leq$ 0.001, 256–600 ms, channels: Fp1, Fp2, F3, F4, F7, F8, Fz, FC1, FC2, FC5, FC6, C3, C4, T7, Cz, FT9, FT10, TP9; negative cluster 2: sum(t): -343, p $\leq$ 0.001, 72–156 ms, channels: Fp1, F3, F7, FC1, FC2, FC5, C3, T7, Cz, FT9. The largest positive cluster is visualized in supplementary figure 1B. All clusters reflect heartbeat independent processes according to a surrogate heartbeat analysis (SFig. 3B, positive-cluster 1: $p = 0.94$, positive-cluster 2: p =1, negative-cluster 1: $p = 0.72$, negative-cluster 2: $p = 1$).

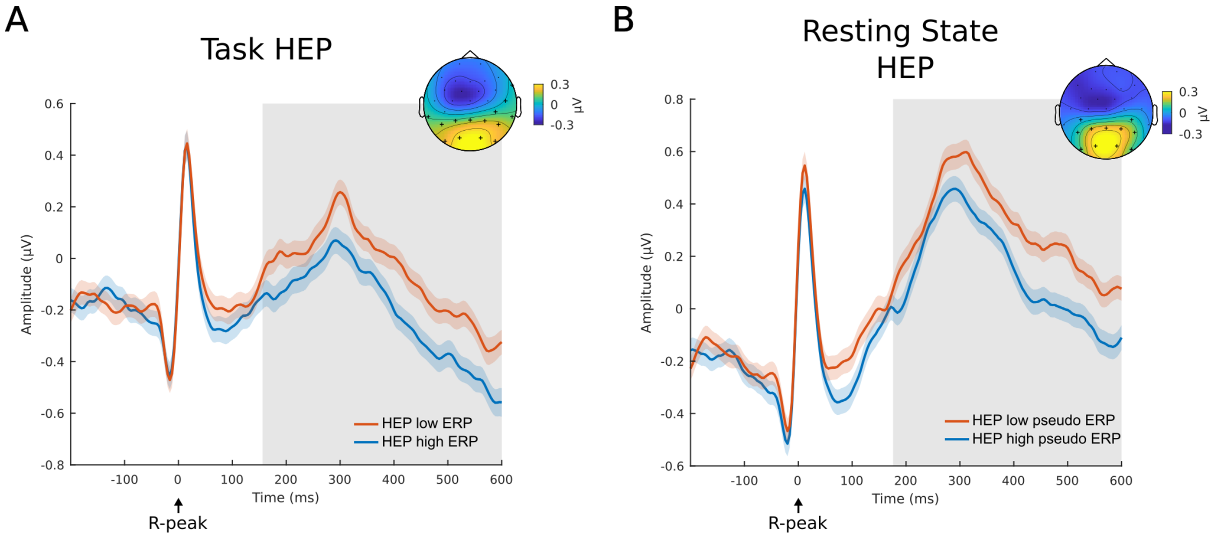

Supplementary Figure 1. HEP differences precede trials sorted in high and low amplitude conditions during task and resting state

A) HEPs preceding target trials sorted into high and low P300 ERPs are significantly different. B) During resting-state, HEPs sorted by the amplitude of "pseudo-ERPs" are significantly different. For resting state and task HEPs the largest positive clusters are shown. Shaded regions around the lines represent ±SEM, gray boxes: p≤0.05, topoplots reflect the average over the largest cluster of significance

with black crosses indicating significant channels.

S2 HEP differences precede ERPs with fast and slow reaction times

In line with the results obtained from baseline-corrected data, we observe significantly different HEPs before trials were sorted into fast and slow reaction time conditions: negative cluster 1: sum(t) = -1494, $p \leq$ 0.001, time: 388 – 600 ms, electrodes: Fz, FC1, FC2, C3, C4, Cz, CP5, CP6, P3, P4, Pz; negative cluster 2: sum(t) = -220, p = 0.012, time: -124 – -80, electrodes: Fp1, Fp2, F7, F8, FC5, T7, FT9, FT10, TP9) and positive cluster 1: sum(t) = 818, $p \leq$ 0.001, time: 464 – 600 ms, electrodes: Fp1, Fp2, F7, F8, FC5, T7, FT9, FT10, TP9; cluster 2: sum(t) = 610, $p \leq$ 0.001, time: -196 – -72 ms, electrodes: F3, Fz, FC1, FC2, FC5, C3, C4, Cz, CP5, CP6, P3, P4, P8, Pz, O2. The largest negative cluster is visualized in supplementary figure 2A. All clusters reflect heartbeat independent processes according to a surrogate heartbeat analysis (SFig. 3C, negative cluster 1: p = 0.87, negative cluster 2: p = 1; positive-cluster 1: p = 0.33, positive-cluster 2: p = 1).

Furthermore, the pre-stimulus time (-200-0 ms) of target trials was significantly different with a similar spatio-temporal distribution to the HEPs (SFig. 2B, cluster sum(t) = -185, $p \leq$ 0.001, electrodes: F4 , Fz , FC1 , FC2 , FC6 , C3 , C4 , Cz , CP5 , CP6, P3 , P4 , Pz).

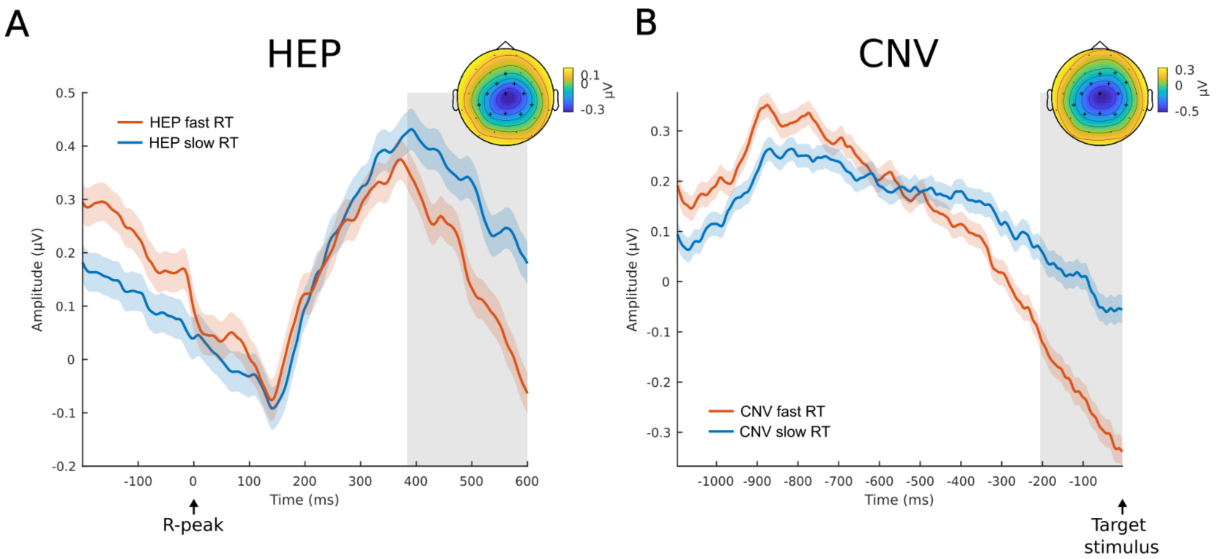

Supplementary Figure 2 Pre-stimulus HEPs sorted by RT during task A) HEP difference before fast and slow reaction time (RT) trials with a late significant negative cluster between 388–600 ms in central electrodes. B) Difference in Contingent Negative Variation (CNV) before fast and slow RT trials. No baseline correction was applied. Shades around the lines are ±SEM. gray boxes: p≤0.05, topoplots reflect the average over largest cluster of significant difference with black crosses indicating significant channels.

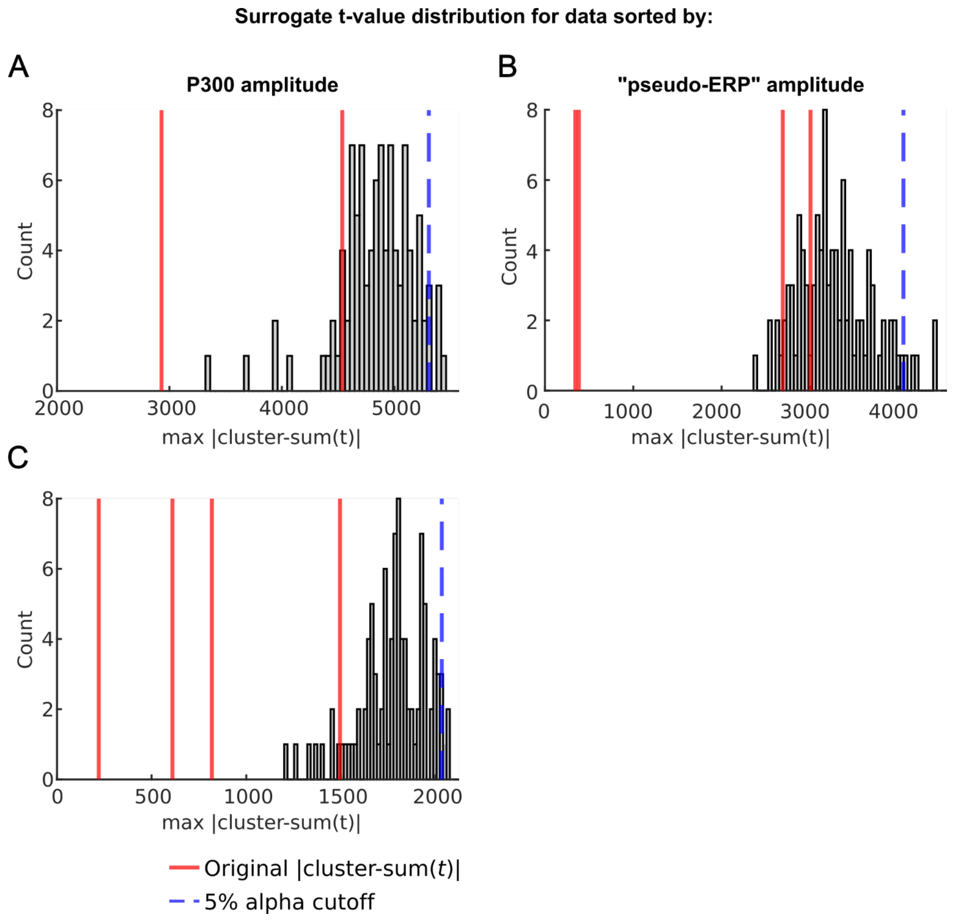

Supplementary Figure 3. Distribution of maximum |cluster-sum(t)|-values obtained from surrogate heartbeat control analyses of non-baseline-corrected HEPs. Histograms illustrate |cluster-sum(*t*)|-values for surrogate heartbeat differences with shuffled R-peak onsets and original HEP effects for all significant clusters (red lines). The related trials are sorted in A) high vs. low P300 amplitude, B) high vs. low "pseudo ERP" amplitude, and C) fast vs. slow reaction time (RT). The dotted blue lines represent the 5% alpha cutoff.

S3 Surrogate heartbeat analysis

In line with the results obtained from baseline-corrected data, none of the clusters reach statistical significance in a surrogate heartbeat control analysis. Histograms in supplementary figure 3 show summed cluster t-values for surrogate HEP differences with shuffled R-peak onsets related to trials sorted in A) high vs. low P300 trials (positive-cluster: $p = 1$, negative-cluster: $p = 0.1$), B) high vs. low "pseudo ERP" trials (positive-cluster 1: $p = 0.94$, positive-cluster 2: $p = 1$, negative-cluster 1: $p = 0.72$, negative-cluster 2: $p = 1$), and C) fast vs. slow RT trials (positive-cluster 1: $p = 0.87$, cluster 2: $p = 1$; negative-cluster 1: $p = 0.87$, $p = 1$). The red lines denote the respective cluster sum(*t*)-values of effects obtained from the original data. The dotted blue lines represent the 5% alpha cutoff. No baseline correction was performed.

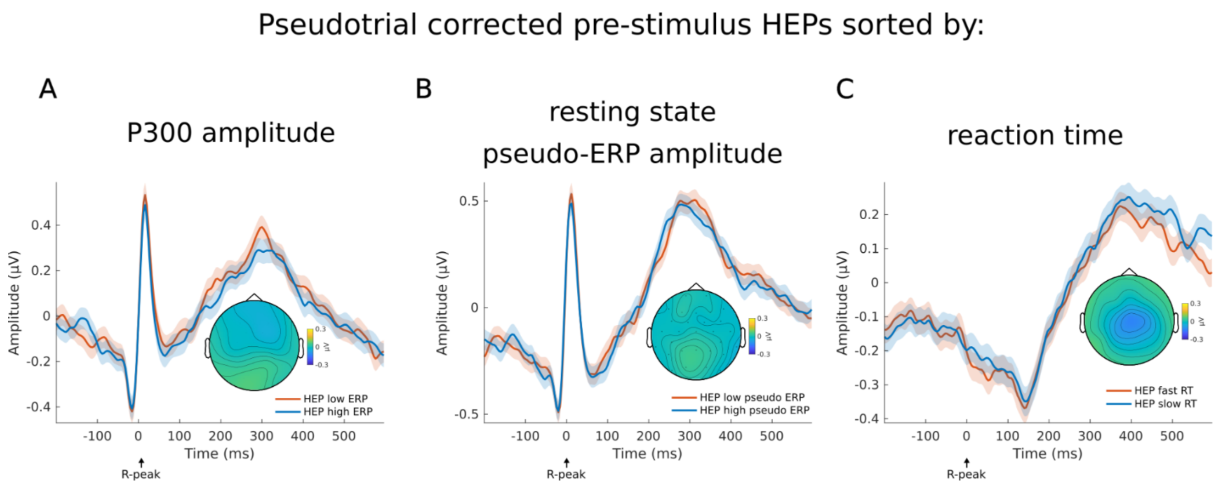

Supplementary Figure 4. No residual pre-stimulus HEP differences after pseudotrial correction

After pseudotrial correction no significant condition differences for the pre-stimulus HEPs between high vs. low P300 ERP amplitude, fast vs. slow reaction times, or pseudo-ERP amplitude during resting state remained. Based on the cluster of significant effects prior pseudotrial correction, HEPs were averaged and compared by equivalence testing. For all three conditions, the absence of a meaningful effect is indicated (HEPs sorted by P300: $t_{1739} = -7.9$, $p < 0.01$; "pseudo ERP" during resting state: $t_{1730} = 7.58$, $p < 0.01$; RT: $t_{1739} = 7.18$, $p < 0.01$).

S4 HEP differences between oddball task and resting state

Before pseudotrial correction, we can only observe one cluster in positive as well as negative direction (SFig. 5A, positive cluster 1: cluster sum(t) = 17343, $p \leq 0.001$, time: -200 – 600 ms, significant channel: Fp1, Fp2, F3, F4, F7, F8, Fz, FC1, FC2, FC5, FC6, C3, C4, T7, Cz, FT9, FT10, CP5, CP6, P3, P4, P8, Pz; negative cluster 1: cluster sum(t): -13498, $p \leq 0.001$, time: -200 – 600 ms, significant channel: Fp1, Fp2, F4, F7, F8, T7, T8, FT9, FT10, CP5, CP6, TP9, TP10, P3, P4, P7, P8, Pz, O1, O2, PO9, PO10). Surrogate heartbeat control analysis (100 iterations) indicates that these clusters were heartbeat unrelated, as all maximum sum(t)-values obtained from shuffled data are larger than the original effects (positive and negative clusters $p = 1$).

Importantly, the heartbeat independent effect can be removed by pseudotrial correction, revealing several clusters distributed in time and space (the first two clusters with the largest sum(t) values are illustrated in SFig. 5B, for a summary of all clusters see supplementary Table 1). Surrogate heartbeat control analysis indicates that the |sum(t)|-values of all clusters fall outside of the 95% percentile range of the cluster |sum(t)|-values obtained from shuffled data (SFig. 5D). Therefore, we can conclude that the effects we observe after pseudotrial correction are coupled to the heartbeat.

To assess the potential influence of cardiac field artifacts on these differences, we compared the ECG between resting state and task by a temporal cluster-based permutation t-test. Several significant differences could be observed (SFig. 5C, largest cluster in positive: cluster sum(t): 952, $p \leq 0.001$, time: 352 - 600 ms, and negative direction: cluster sum(t): -597, $p \leq 0.001$, time: 216 - 344 ms, all other clusters: $p \leq 0.001$), indicating potential differences in CFA when comparing resting state and task HEPs. To further investigate if the CFA can explain the HEP differences, we tested whether the difference between resting state and task ECG (ΔECG correlates to the difference between resting state and task EEG (ΔEEG. No significant clusters were found in the HEP time window of interest (SFig. 6).

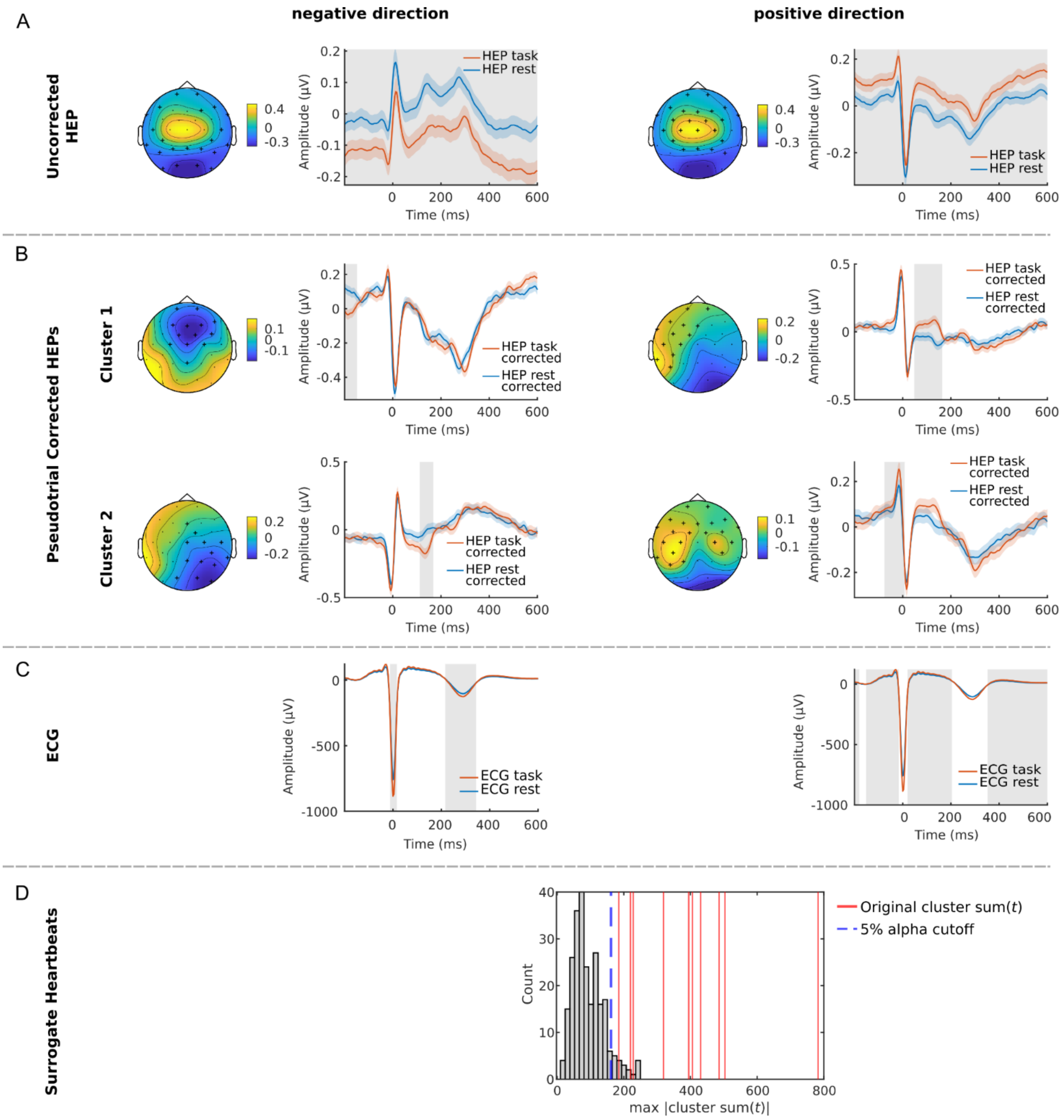

Supplementary Figure 5. HEP differences between oddball task and resting state for non- baseline-corrected data Differences in HEPs between Oddball task and resting state in positive and negative direction A) before and B) after pseudotrial correction. After correction, several clusters were found and the strongest first two are illustrated. C) ECG differences between task and rest are illustrated. Shades around the lines are ±SEM, grey boxes: $p \leq 0.05$, Topoplots are averaged over time of significant differences with crosses illustrating significant differences. D) Histogram visualizing the surrogate heartbeat control analysis. All clusters of the original data have larger absolute cluster sum(t)-values (red lines) than the 5% alpha cutoff (blue dashed lines) obtained from the maximum surrogate statistics (gray bars).

Supplementary Table 1. Significant differences between resting state and. task related HEPs after pseudotrial correction - non-baseline corrected data

Direction	*p*-value	sum(t)-value	Time Range (ms)	Electrodes in Cluster
positive	≤ 0.001	782.82	[48,164]	Fp1, Fp2, F3, F7, Fz, FC1, FC5, C3, T7, FT9, CP5, TP9, P7
positive	≤ 0.001	503.37	[-76,8]	Fp1, Fp2, F3, F4, F7, F8, Fz, FC1, FC2, FC5, FC6, C3, C4, T7, T8, FT9, FT1,CP5, CP6, TP9, P3, P7
positive	≤ 0.001	405.94	[532,600]	Fp1, Fp2, F3, F4, F8, Fz, FC1, FC2, FC5, FC6, C4, T8, Cz, FT1,TP10
positive	≤ 0.001	319.04	[-200,-156]	T7, FT9, CP5, TP9, TP1,P3, P7, P8, O1, O2, PO9, PO10
negative	≤ 0.001	-486	[-200,-148]	Fp1, Fp2, F3, F4, F8, Fz, FC1, FC2, FC6, C3, C4, Cz, Pz
negative	≤ 0.001	-430.41	[112,168]	Fz, FC2, C4, T8, Cz, CP6, TP1,P4, P8, Pz, O1, O2, PO10
negative	≤ 0.001	-394.48	[180,280]	F3, Fz, FC1, FC2, C3, C4, T8, Cz, CP5, CP6, TP1,P3, P4, P8, O2, PO10
negative	0.008	-228.2	[560,600]	C3, T7, CP5, CP6, TP9, P3, P4, P7, Pz, O1
negative	0.01	-219.99	[-24,20]	Fp1, F7, FC5, T7, FT9, TP9, P4, P7, P8, Pz, O1, O2, PO9, PO10
negative	0.023	-185.04	[292,324]	F3, F4, Fz, FC1, FC2, FC6, C4, Cz

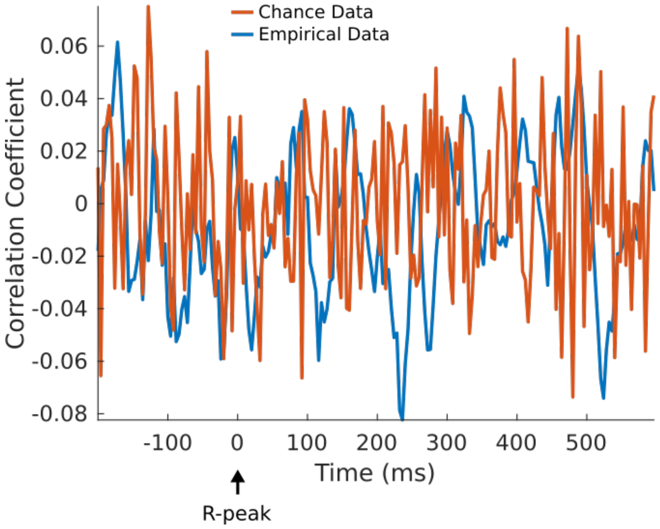

Supplementary Figure 6. No Significant Correlation between ΔECG and ΔEEG in time range of HEP analysis Correlation between resting-state and task-related differences in ECG (ΔECG) and EEG (ΔEEG) across all channels and time points (Empirical Data) and corresponding chance data obtained by correlation of ΔEEG with randomized ΔECG data. No clusters of significant differences were found (p>0.05). Time courses for illustration are obtained from the Cz electrode and grand averaged across subjects. No baseline correction was performed.

Supplementary Table 2. Clusters of significant differences between resting state and task HEPs after pseudotrial correction - baseline corrected data

Direction	*p*-value	sum(*t*)-value	Time Range (ms)	Electrodes in Cluster
positive	≤ 0.001	414.13	[476, 556]	Fp1, Fp2, F3, F4, F7, F8, Fz, FC2, FC6, Cz, FT10
positive	≤ 0.001	385.14	[104, 160]	Fp1, Fp2, F3, F4, F7, F8, Fz, FC1, FC2, FC5, T7, FT9, TP9
positive	0.007	274.91	[-24, 12]	Fp1, Fp2, F3, F4, F7, F8, Fz, FC1, FC2, FC5, FC6, C3, C4, T7, T8, FT10
positive	0.017	227.5	[568, 600]	Fp1, Fp2, F3, F4, F8, Fz, FC2, FC6, Cz, FT10
negative	≤ 0.001	-911.71	[368, 600]	F3, FC1, FC5, C3, T7, FT9, CP5, CP6, TP9, P3, P4, P7, P8, Pz
negative	≤ 0.001	-616.67	[112, 240]	FC1, C3, C4, T8, Cz, CP5, CP6, TP10, P3, P4, P7, P8, Pz, O1, O2, PO10
negative	0.003	-271.89	[-200, -160]	F3, F4, Fz, FC1, FC2, FC6, C3, C4, Cz
negative	0.004	-249.27	[-20, 24]	F7, FC5, C3, T7, FT9, CP5, TP9, P3, P7, P8, O1, O2, PO9, PO10
negative	0.023	-187.81	[260, 332]	F3, FC1, C3, CP5, TP9, P7

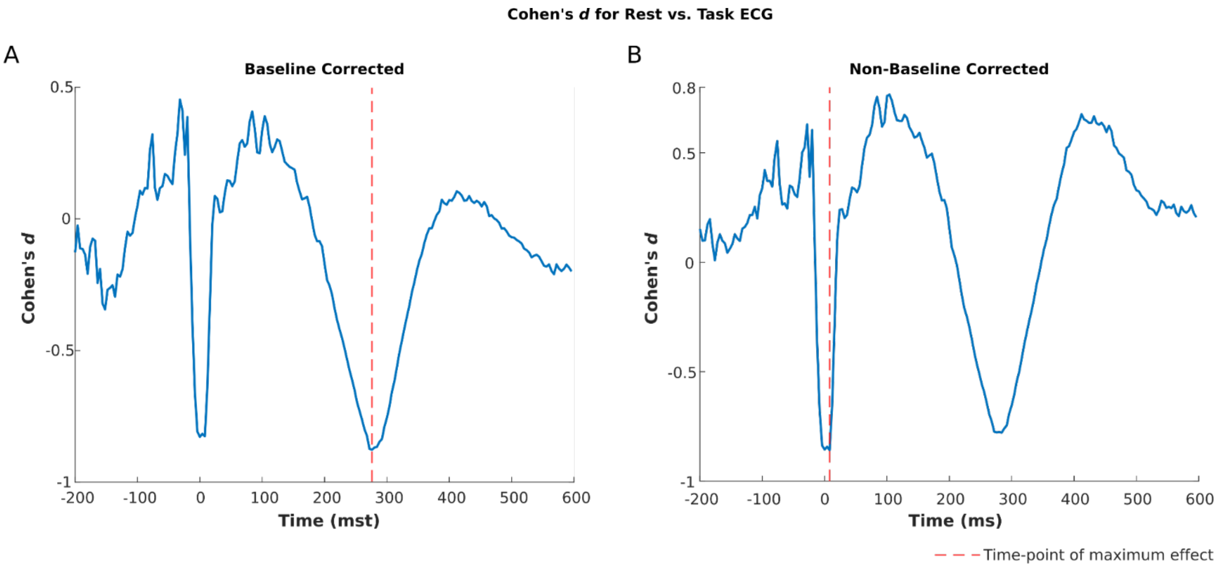

Supplementary Figure 7. Effect size of ECG difference between Oddball task and resting state. Time series illustrating the temporal evolution of Cohen's *d* for the difference in ECG signals between resting-state and oddball task conditions. Baseline-corrected (A) and non-baseline-corrected (B) data are shown. The largest effect sizes (red dashed lines) occur at 276 ms (baseline-corrected; Cohen's d = –0.876, coinciding with the T-wave) and 8 ms (non-baseline-corrected; Cohen's d = -0.858, coinciding with the R-peak).
